# Mechanical circulatory support for infarct-related cardiogenic shock: a systematic review, pairwise and network meta-analysis

**DOI:** 10.1093/ehjopen/oeaf091

**Published:** 2025-07-29

**Authors:** Zaran Butt, Saad Sharif, Mohammed Ahmad, Michael J Daly, James O’Neill, Aleksandra Gentry-Maharaj, Peter J Godolphin

**Affiliations:** MRC Clinical Trials Unit, Institute of Clinical Trials and Methodology, University College London, WC1V 6LJ London, UK; Department of Cardiology, Connolly Hospital, Blanchardstown, D15 X40D Dublin, Ireland; Department of Cardiology, Connolly Hospital, Blanchardstown, D15 X40D Dublin, Ireland; Department of Cardiology, Connolly Hospital, Blanchardstown, D15 X40D Dublin, Ireland; Department of Cardiology, Connolly Hospital, Blanchardstown, D15 X40D Dublin, Ireland; Royal College of Surgeons in Ireland, University of Medicine and Health Sciences, 123 St. Stephen’s Green, D02 YN77 Dublin, Ireland; Department of Cardiology, Connolly Hospital, Blanchardstown, D15 X40D Dublin, Ireland; Royal College of Surgeons in Ireland, University of Medicine and Health Sciences, 123 St. Stephen’s Green, D02 YN77 Dublin, Ireland; MRC Clinical Trials Unit, Institute of Clinical Trials and Methodology, University College London, WC1V 6LJ London, UK; Department of Women’s Cancer, EGA Institute for Women’s Health, University College London, WC1E 6DE London, UK; MRC Clinical Trials Unit, Institute of Clinical Trials and Methodology, University College London, WC1V 6LJ London, UK

**Keywords:** Cardiogenic shock, Mechanical circulatory support, Veno-arterial extra-corporeal membrane oxygenation, Percutaneous ventricular assist device, Intra-aortic balloon pump, Myocardial infarction

## Abstract

**Aims:**

Mortality from cardiogenic shock complicating acute myocardial infarction (AMI-CS) remains high, despite the increasing mechanical circulatory support (MCS) use in clinical practice.

**Methods and results:**

We undertook a systematic review and meta-analysis of trials assessing MCS in adults with AMI-CS. We searched Medline, EMBASE, CENTRAL, Web of Science, and Scopus from inception to May 2024. We evaluated the effect of each intervention on early mortality using a random-effects network meta-analysis of odds ratios (ORs). Safety outcomes included stroke, bleeding, and sepsis. Fourteen trials randomizing 1858 patients were included: intra-aortic balloon pump (IABP) vs. medical therapy (four trials, *n* = 748 patients), veno-arterial extra-corporeal membrane oxygenation (VA-ECMO) vs. No VA-ECMO (four trials, *n* = 568 patients), percutaneous ventricular assist device (pVAD) vs. No pVAD (six trials, *n* = 542 patients). No MCS device showed a significant effect on early mortality vs. initial medical therapy {IABP (OR 0.87, 95% CI 0.66–1.15), VA-ECMO (OR 0.91, 95% CI 0.65–1.27), pVAD (OR 0.80, 95% CI 0.56–1.14), and *P* (inconsistency) = 0.76}. VA-ECMO and pVAD were associated with increased major bleeding [OR 2.81 (95% CI 1.68–4.71) and OR 5.13 (95% CI 1.87–14.04), respectively]. Higher rates of stroke and sepsis were noted with pVAD. No significant safety concerns were identified with IABP.

**Conclusion:**

The mortality benefit of MCS devices in AMI-CS remains uncertain. Using such devices may be associated with increased risks, including major bleeding, stroke, and sepsis. Current evidence does not support the routine use of MCS devices in the management of AMI-CS.

## Graphical Abstract

**Graphical Abstract:**
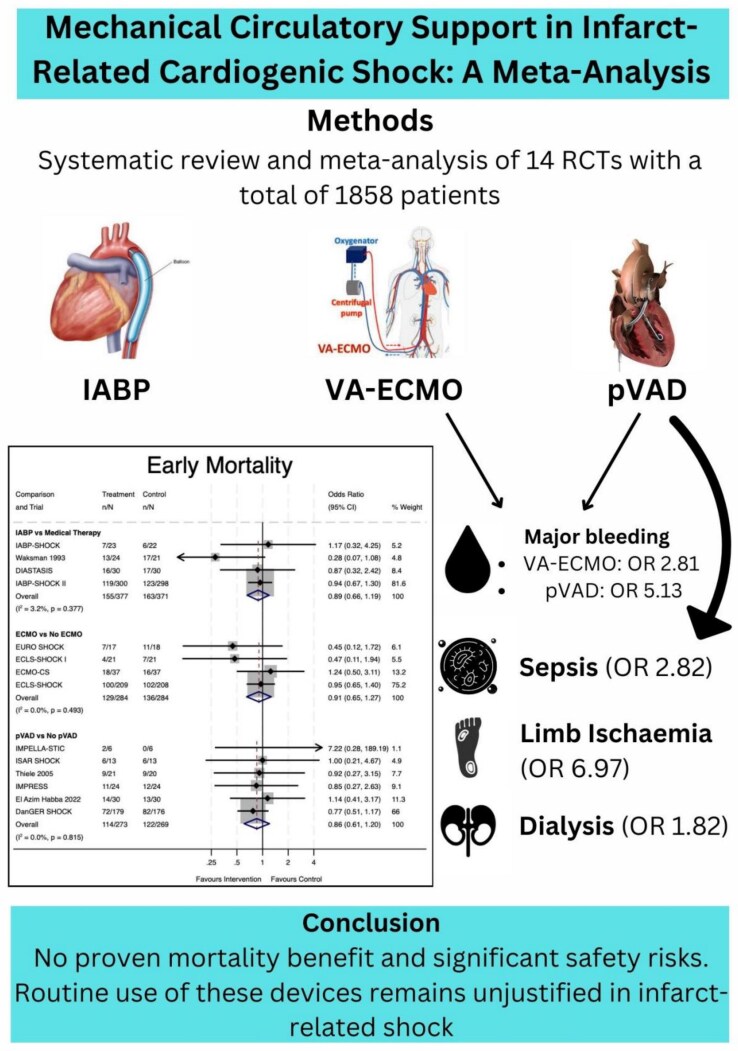
Mechanical circulatory support in infarct-related shock. IABP, intra-aortic balloon pump; VA-ECMO, veno-arterial extra-corporeal membrane oxygenation; pVAD, percutaneous ventricular assist device; OR, odds ratio; RCT, randomized controlled trial.

## Introduction

Cardiogenic shock is a potentially catastrophic complication of acute myocardial infarction (AMI) and occurs in up to 10% of cases.^1^ Early mortality for those who develop AMI-related cardiogenic shock (AMI-CS) remains significant despite overall improvements in AMI-related mortality.^1^ In recent decades, a number of temporary mechanical circulatory support (MCS) devices have been developed in an attempt to improve the prognosis of patients with AMI-CS.^2^ These include intra-aortic balloon pumps (IABP), veno-arterial extra-corporeal membrane oxygenation (VA-ECMO), and percutaneous ventricular assist devices (pVAD). However, their efficacy in AMI-CS remains controversial, along with considerable economic implications and potential device-related risks, i.e. vascular damage, bleeding, cerebrovascular accidents, and limb ischaemia.^2^

Positive efficacy data for MCS use in AMI-CS is mainly derived from observational studies^3,4^; however, these observational studies were likely under-powered and most pre-date the era of routine early revascularization in AMI-CS, i.e. the only intervention shown to improve mortality in these patients.^5^ Despite this limited evidence base and a Class IIb recommendation for their use in AMI-CS in contemporary guidelines,^6^ clinical use of MCS devices in this setting has increased exponentially over the past decade in Western countries.^7^ IABPs are the most popular device, used in up to 40% of AMI-CS cases in the USA.^7^ Randomized controlled trials have shown no 30-day mortality benefit for MCS devices in AMI-CS; however, no single trial was sufficiently powered to detect a treatment effect.^8–13^

The last network meta-analysis of temporary MCS in AMI-CS found no in-hospital or 30-day mortality benefit over medical therapy with any device, either singularly or in combination.^14^ That analysis may have been biased by a non-systematic search strategy and investigators did not report on safety outcomes, which are relevant to the considerable device-related risks associated with MCS devices.^2^ Furthermore, two landmark trials have since been published, representing the largest trials investigating pVAD and VA-ECMO in AMI-CS to date.^15,16^ The DanGer Shock investigators enrolled 355 patients and found a statistically significant 180-day mortality benefit with pVAD use compared to standard care [hazard ratio 0.74, 95% confidence interval (CI) 0.55–0.99, *P* = 0.04].^15^ By contrast, ECLS-Shock randomized 417 patients with AMI-CS planned for early revascularization and reported no 30-day mortality benefit with VA-ECMO compared to standard care (relative risk 0.98; 95% CI 0.80–1.19, *P* = 0.81).^16^

The primary aim of this study is to determine whether temporary MCS improves early mortality in adults with AMI-CS. The objective of this study was to complete an updated systematic review and network meta-analysis, providing a contemporary comparative assessment of the evidence from randomized trials on different MCS devices. As such, this study might highlight which, if any, MCS devices warrant prioritization for future trials.

## Methods

This systematic review and meta-analysis was reported according to the preferred reporting items for systematic reviews and meta-analyses (PRISMA) guidelines (see Supplementary material online, *Table S1*).^17^ The study was prospectively registered on the PROSPERO international register of systematic reviews (Study ID: CRD42024546141). Patients and Public were not involved in any aspect of this work.

### Eligibility criteria and search strategy

Eligible studies were randomized-controlled trials investigating the use of temporary MCS in adults with infarct-related cardiogenic shock planned for early revascularization (primary percutaneous coronary intervention (PCI) or coronary artery bypass grafting). Trials with heterogeneous control arms were included due to the lack of an accepted universal standard of care and common use of MCS devices in clinical practice.

The search strategy was developed using the ‘PICOS’ framework outlined in Supplementary material online, *Table S2*. Search concepts and key words were identified using a combination of key paper screening and a review of search strategies from previous reviews.

The following databases were searched from inception to 20 May 2024 (search strategies available in appendix): Medline, EMBASE, CENTRAL, Web of Science, and Scopus. A grey literature search of key trial registries (ClinicalTrials.gov, ISRCTN) was performed to supplement the bibliographic database search.

### Selection of studies and data extraction

Identified articles were de-duplicated using Systematic Review Accelerator, before title and abstract screening using the same software. Full-text screening of remaining articles was completed using Endnote version 20.5. Articles not in English and those without full-text availability were excluded.

Data were extracted from selected articles using a pre-designed data extraction sheet. Two independent reviewers conducted screening, with discordances resolved by a third independent third reviewer. A single reviewer conducted all risk-of-bias assessments and data extraction.

### Outcomes

The primary outcome for was all-cause early mortality (30-day or in-hospital mortality). A pre-specified secondary efficacy outcome of late (1 year) all-cause mortality was also planned depending on availability of data.

Pre-specified safety outcomes included the following (defined as per author’s definitions): Initiation of urgent renal replacement therapy, acute limb ischaemia, stroke, length of hospital stay, major bleeding, and major vascular complications.

Odds ratios (OR) were used as the effect measure for each outcome, with unadjusted OR’s estimated from trials based on the total number of events and participants in each arm. No missing data was imputed for any analysis. The number needed to treat or number needed to harm (NNH) was calculated for any statistically significant results. All statistical analysis was completed using Stata Version 18.2 or later.

### Quality assessment

A risk-of-bias assessment for the primary outcome of early mortality was completed for all individual studies using the Cochrane risk-of-bias 2 tool.^18^ Additionally, the Critical Appraisal Skills Programme checklist for randomized trials was used to guide appraisal of individual studies.^19^

### Meta-analysis

Included trials were sub-categorized into one of three possible comparisons based on intervention arm: IABP vs. medical therapy; VA-ECMO vs. No VA-ECMO; and pVAD vs. No pVAD.

Pairwise meta-analyses were performed using inverse-variance random-effects models (restricted maximum likelihood estimator of Tau^2^) for each comparison to estimate an overall treatment effect for each individual intervention, with results displayed using forest plots. Inconsistency between trials for each direct comparison was assessed using the *I*^2^ statistic, and Cochran’s Q test and visual inspection of forest plots were used to assess heterogeneity. For the pairwise meta-analysis, a trial-level subgroup analysis by region (Europe vs. non-European) was planned, along with two sensitivity analyses: (i) using relative risk as an effect measure instead of OR, and (ii) excluding studies at high risk of bias for early mortality. In addition, we performed the following *post-hoc* analyses to address clinical heterogeneity between the included trials: trial-level subgroup analyses (i) Single-centre/multi-centre trials, and (ii) Impella devices vs. TandemHeart for the pVAD vs. No pVAD comparison, and sensitivity analyses (i) limited to studies at low risk of bias for early mortality, (ii) excluding studies with >15% cross-over rate, and (iii) limited to studies with 0% cross-over rate.

A network meta-analysis of ORs was used to estimate the effect of each intervention on early mortality, using a frequentist arm-based approach that implemented random-effects multivariate meta-analysis models that assumed consistency between direct and indirect evidence.^20^ This assumption was tested using a design-by-treatment interaction model.^20^ We calculated borrowing of strength statistics using the score decomposition method^18^ to demonstrate the weight of information for each network OR that is due to indirect evidence. Trials that assessed combinations of interventions (e.g. pVAD and IABP) were excluded from the primary network but included in sensitivity analyses as a separate node. This was fitted using the network suite of packages in Stata.

### Patients and public involvement

Patients were not involved in any aspect of this work.

## Results

### Search results

**Figure 1:**
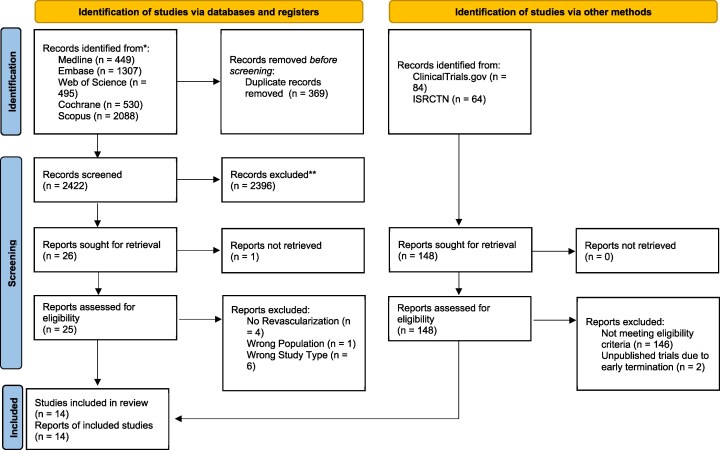
PRISMA flow diagram.^17^ Total of 11 articles was excluded after full-text screening; four lacked an early coronary revascularization strategy, one did not include patients with infarct-related shock, while six were not randomized-controlled trials.

The search strategy for this study is summarized in a PRISMA flow diagram (*Figure 1*). A total of 2422 articles were screened from five databases. Following de-duplication and title/abstract screening, 26 articles underwent full-text screening. No full-text was available for one small IABP trial published in 1969, which randomized 29 patients.^19^ Of those remaining (*n* = 25), 14 trials, which randomized a total of 1858 patients were deemed eligible for inclusion.^3,8-13,15,16,21-25^ The grey literature search yielded two unpublished trials; however, a full-text report was unavailable for both.

### Study characteristics

Important study characteristics are summarized in *Table 1*. Most trials were conducted in Europe (71%) and the majority (57%) were multi-centre trials. Trials were sub-categorized based on intervention group: IABP (*n* = 4 trials); VA-ECMO (*n* = 4 trials); and, pVAD (*n* = 6 trials). From the pVAD studies, 74% of patients randomized to intervention across the trials received an Impella CP device. Alternative micro-axial flow pumps such as Impella 2.5 (13%), Impella 5.0 (2%), and TandemHeart (8%) were used as the primary device in remaining cases. The combined sample sizes were 748, 568, and 542 patients for IABP, VA-ECMO, and pVAD trials, respectively. Studies were published between 1993 and 2024. All trials were open-label, had 1:1 parallel randomization, and used an intention-to-treat or modified intention-to-treat primary analysis set. In-hospital mortality was reported in three trials,^3,21,22^ while the remaining reported 30-day mortality data. Cross-over from control to intervention therapies was particularly high in ECMO-CS (39%), which permitted cross-over if there was a serum lactate rise >3 mmol/L within a 24-h period.^9^ Definitions of cardiogenic shock were similar across trials; apart from, IMPRESS^11^ which incorporated a requirement for mechanical ventilation, and IMPELLA-STIC,^12^ which incorporated IABP use. A total of five (36%) trials were deemed to have a high overall risk of bias for the primary outcome of early mortality, while only two (14%) were deemed to be of low overall risk. Bias was mostly attributable to the randomization process or deviations from the intended intervention (see Supplementary material online, *Table S3*).

**Table 1:** Overall trial comparisons

Trial	Countries (sites)	Sample Size	Primary MCS	Control	MCS pre-PCI (%)	MCS post-PCI (%)	Cross-over rate (%)	Cardiogenic shock severity	RoB
Intra-aortic balloon pump:									
Prondzinsky *et al.* 2010	Germany (1)	45	IABP	Medical Therapy	0	100	5	Symptoms and signs of organ hypo-perfusion (cool peripheries, oliguria) AND one of: SBP <90 mmHg for at least 30 min, hypotension requiring inotropic/vasopressor therapy at a heart rate >60 b.p.m. or a cardiac index <2.2 L/min/m^2^ on invasive monitoring	Low
Sharma *et al.* 2022	India (1)	60	IABP	Medical therapy	100	0	0	SBP <90 mmHg for >30 min or required catecholamine infusions to maintain SBP >90, and clinical signs of pulmonary congestion, AND impaired end-organ perfusion (at least one of: Altered mental status, cold clammy skin, and extremities, oliguria with urine output <30 mL/h, lactate >2 mmol/L)	High
Thiele *et al.* 2012	Germany (37)	598	IABP	Medical therapy	13	87	10	SBP <90 mmHg for >30 min OR required catecholamine infusions to maintain SBP >90, and clinical signs of pulmonary congestion, AND impaired end-organ perfusion (at least one of: Altered mental status, cold clammy skin, and extremities, oliguria with urine output <30 mL/h, lactate >2 mmol/L)	Some
Waksman *et al.* 1993	Israel (1)	45	IABP	Medical therapy	100	0	0	SBP <90 mmHg refractory to fluid challenge + urine output <20 mL/h + ‘peripheral evidence of circulatory failure’	High
Veno-arterial extra-corporeal membrane oxygenation:									
Ostadal *et al.* 2023	Czech Republic (4)	74	VA-ECMO	Medical therapy	–	–	39	Rapidly deteriorating cardiogenic shock: progressive haemodynamic instability necessitating repeated bolus administration of vasopressors to maintain mean arterial pressure >50 mmHg + impaired LVEF (<35% or 35–55% with severe MR or AS) Severe cardiogenic shock: haemodynamic criteria [cardiac index <2.2 L/min/m^2^ AND (Noradrenaline >0.1 µg/kg/min OR dobutamine >5 µg/kg/min], OR SBP <100 mmHg AND (Noradrenaline >0.2 µg/kg/min OR dobutamine >5 µg/kg/min) and (LVEF <35% OR LVEF 35–55% with severe MR or AS)] and metabolic criteria (two consecutive lactate >3 mmol/L with non-decreasing trend on steady doses of inotropes/vasopressors or two consecutive mixed venous oxygen saturations <50% with non-increasing trend on steady doses of inotropes/vasopressors) AND Exclusion of hypovolemia (central venous pressure >7 mmHg OR pulmonary capillary wedge pressure >12 mmHg)	High
Banning *et al.* 2023	Spain, Germany, UK, Norway, Latvia, Belgium (15)	35	VA-ECMO	Medical Therapy	0	100	6	SBP <90 mmHg for >30 min or required catecholamine infusions to maintain SBP >90, and clinical signs of pulmonary congestion, and impaired end-organ perfusion (at least one of: Altered mental status, cold clammy skin, and extremities, oliguria with urine output <30 mL/h, lactate >2 mmol/L)	Some
Brunner *et al.* 2019	USA (1)	42	VA-ECMO	Medical Therapy	–	–	–	SBP <90 mmHg for >30 min or required catecholamine infusions to maintain SBP >90, and clinical signs of pulmonary congestion, AND impaired end-organ perfusion (at least one of: Altered mental status, cold clammy skin, and extremities, oliguria with urine output <30 mL/h, lactate >2 mmol/L)	High
Thiele *et al.* 2023	Germany, Slovenia (44)	417	VA-ECMO	Medical therapy	48	52	12.5	SBP <90 mmHg for >30 min or required catecholamine infusions to maintain SBP >90, and clinical signs of pulmonary congestion, and impaired end-organ perfusion (at least one of: Altered mental status, cold clammy skin, and extremities, oliguria with urine output <30 mL/h, lactate >3 mmol/L)	Some
Percutaneous ventricular assist devices:									
Bonnefoy-Cudraz *et al.* 2014	France (2)	12	IMPELLA 5.0 & IABP	IABP	0	100	0	Requiring inotropic drugs and an intra-aortic balloon pump	Some
Moller *et al.* 2024	Denmark, Germany, UK (3)	355	IMPELLA CP	Medical Therapy	47	53	1.7	SBP <100 mmHg or ongoing need for vasopressors, and end-organ hypo-perfusion with lactate ≥2.5 mmol/L, and LVEF <45%	Some
Seyfarth *et al.* 2008	Germany (2)	26	IMPELLA 2.5	IABP	0	100	0	Clinical {(SBP <90 for >30 min or need for inotropes/vasopressors to maintain SBP >90) and clinical signs of end-organ hypo-perfusion (cool extremities or urine output <30 mL/h) AND (heart rate > 60 b.p.m.)} and haemodynamic criteria {cardiac index <2.2 L/min/m^2^ and (pulmonary capillary wedge pressure >/= 15 mmHg or signs of pulmonary congestion on chest x-ray if anterior infarction)}	Some
Ouweneel *et al.* 2017	Holland, Norway (2)	48	IMPELLA CP	IABP	20	80	13	SBP <90 mmHg for >30 min or required catecholamines to maintain SBP >90 mmHg, and required mechanical ventilation	Some
El Azim Habba *et al.* 2022	Kuwait (1)	60	IMPELLA CP	IABP	–	–	16.7	SBP <90 mmHg or required catecholamines, and ‘impaired perfusion’	High
Thiele *et al.* 2005	Germany (1)	41	Tandem Heart	IABP	57	43	0	SBP <90 mmHg or vasopressors required to maintain SBP >90 mmHg, and evidence of end-organ failure (urine output <30 mL/h, cold extremities, lactate >2 mmol/L), and evidence of elevated left ventricular filling pressures (‘pulmonary congestion’ OR pulmonary capillary wedge pressure >15 mmol/L AND cardiac index <2.1 L/min/m^2^)	Low

‘Risk of bias’ represents overall risk of bias of each trial with respect to the outcome of early mortality only, assessed using the revised Cochrane risk-of-bias tool for randomized trials.^26^ Cross-over rate refers to cross-over from control to intervention arm and is expressed as a percentage of the control arm sample. MCS before or after PCI refers to timing of MCS insertion relative to timing of coronary revascularization in the intervention arm in each trial.

UK, United Kingdom; SBP, systolic blood pressure; LVEF, left ventricular ejection fraction; MR, mitral regurgitation; AS, aortic stenosis; MCS, mechanical circulatory support; RoB, risk of bias; PCI, percutaneous coronary intervention; CABG, coronary artery bypass graft.

Relevant baseline characteristics in the control and intervention arms of each trial are summarized in *Table 2*. Average age across trials ranged from 53 to 70 years, with men the majority of participants in all studies. Average systolic blood pressure (SBP) ranged from 78 to 106 mmHg, average left ventricular ejection fraction (LVEF) from 20% to 35% and average lactate from 1.8 to 9.0 mmol/L. High rates (>85%) of early revascularization with PCI was evident in all but two trials; ECMO-CS,^9^ which enrolled an undifferentiated cardiogenic shock population with 62.4% having infarct-related shock (>85% PCI rate in this subgroup), and Waksman *et al*.^3^ where only 38% of patients received PCI with a heavily skewed distribution in favour of the intervention arm (16:1). Other pre-defined prognostically relevant baseline characteristics could not be directly compared across trials due to missing data or heterogeneous reporting.

**Table 2:** Key baseline characteristics are presented for intervention (*_i_*) and control (*_c_*) arms in each trial

Trial	Age*_i_* (years)	Age*_C_*	Male*_i_* (%)	Male*_C_*	MV*_i_* (%)	MV*_C_*	CPR*_i_* (%)	CPR*_C_*	SBP*_i_* (mmHg)	SBP*_C_*	LVEF*_I_* (%)	LVEF*_C_*	Lactate*_i_* (mmol/L)	Lactate*_C_*
Intra-aortic balloon pump:														
Prondzinsky *et al.* 2010	62.1 (38–82)	66.1 (49–82)	74	81	37	67	–	–	–	–	–	–	–	–
Sharma *et al.* 2022	60 (50–83)	57 (48–75)	70	76.7	–	–	–	–	80 (71.5–80)	78 (71.5–86)	30 (23.8–20)	25 (20–30)	4.0 (2.6–6.9)	4.8 (2.72–9.75)
Thiele *et al.* 2012	70 (58–78)	69 (58–76)	67.1	70.6	79.7	84.3	42.2	47.8	89 (79–107)	90 (80–109)	35 (25–45)	35 (24–45)	3.6 (2.1–7.2)	4.7 (2.3–8.2)
Waksman *et al.* 1993	66.8 (±11.4)	67.8 (± 13.2)	58	67	33	33	71	38	85 (±10)	85 (±10)	–	–	–	–
Veno-arterial extra-corporeal membrane oxygenation:														
Ostadal *et al.* 2023	67 (60–74)	65 (58–71)	74.1	72.9	–	70.2	–	–	84.0 (80–95)	89 (79.5–105.0)	–	–	5.3 (3.1–8.4)	4.7 (3.3–7.4)
Banning *et al.* 2023	68 (60–73)	67 (56–77)	81	89	67	75	53	44	82 (75–105)	95 (81–125)	20 (10–35)	25 (15–35)	5.9 (±3.7)	8.2 (±4.6)
Brunner *et al.* 2019	62 (50–68)	70 (60–74)	–	–	–	–	–	–	–	–	–	–	4.8 (2.7–8.5)	5.4 (3.3–9.2)
Thiele *et al.* 2023	62 (56–69)	63 (57–71)	81.3	81.2	90.1	87.6	77.5	77.9	95 (80–120)	97 (80–120)	30 (20–35)	30 (20–40)	6.8 (4.5–9.6)	6.9 (4.6–10.0)
Percutaneous ventricular assist device:														
Bonnefoy-Cudraz *et al.* 2014	53.5 (±8.1)	60.3 ± 12.3	100	85.7	0	–	–	–	–	–	29.3 (±6.7)	29.7 (±8.4)	1.3 (±0.3)	1.7 (±0.4)
Moller *et al.* 2024	67 (58–76)	69 (61–76)	79.3	79	74.3	53.4	21.8	18.8	84 (72–91)	82 (72–91)	25 (20–31)	25 (15–30)	4.6 (3.4–7.1)	4.5
Seyfarth *et al.* 2008	65 (57–71)	67 (55–80)	62	85	92	69	–	–	106 (±22)	101 (±23)	27 (20–39)	28 (33–44)	–	–
Ouweneel *et al.* 2017	58 (±9)	59 ± 11	75	83	100	29	100	83	81 (±17)	84 (±19)	–	–	7.5 (±3.2)	8.9 (±6.6)
El Azim Habba *et al.* 2022	58.9 (±11)	56 (±9)	86.7	90	73.3	66.7	30	16.7	81 (±9.6)	81.2 (±3.7)	25 (±10)	30 (±8.2)	9 (±3)	6.7 (±3.5)
Thiele *et al.* 2005	63 (57–70)	65 (59–73)	76	75	95	75	–	–	–	–	25 (20–32.8)	28.5 (20.5–30.5)	4.5 (3.1–6.5)	3.8 (3.5–6.7)

Continuous variables are expressed as mean (±standard deviation) or median (inter-quartile range). Categorical variables are expressed as percentages.

HT, hypertension; DM, diabetes mellitus; SBP, systolic blood pressure; LVEF, left ventricular ejection fraction; MV, mechanical ventilation; CPR, cardiopulmonary resuscitation.

### Efficacy

**Figure 2:**
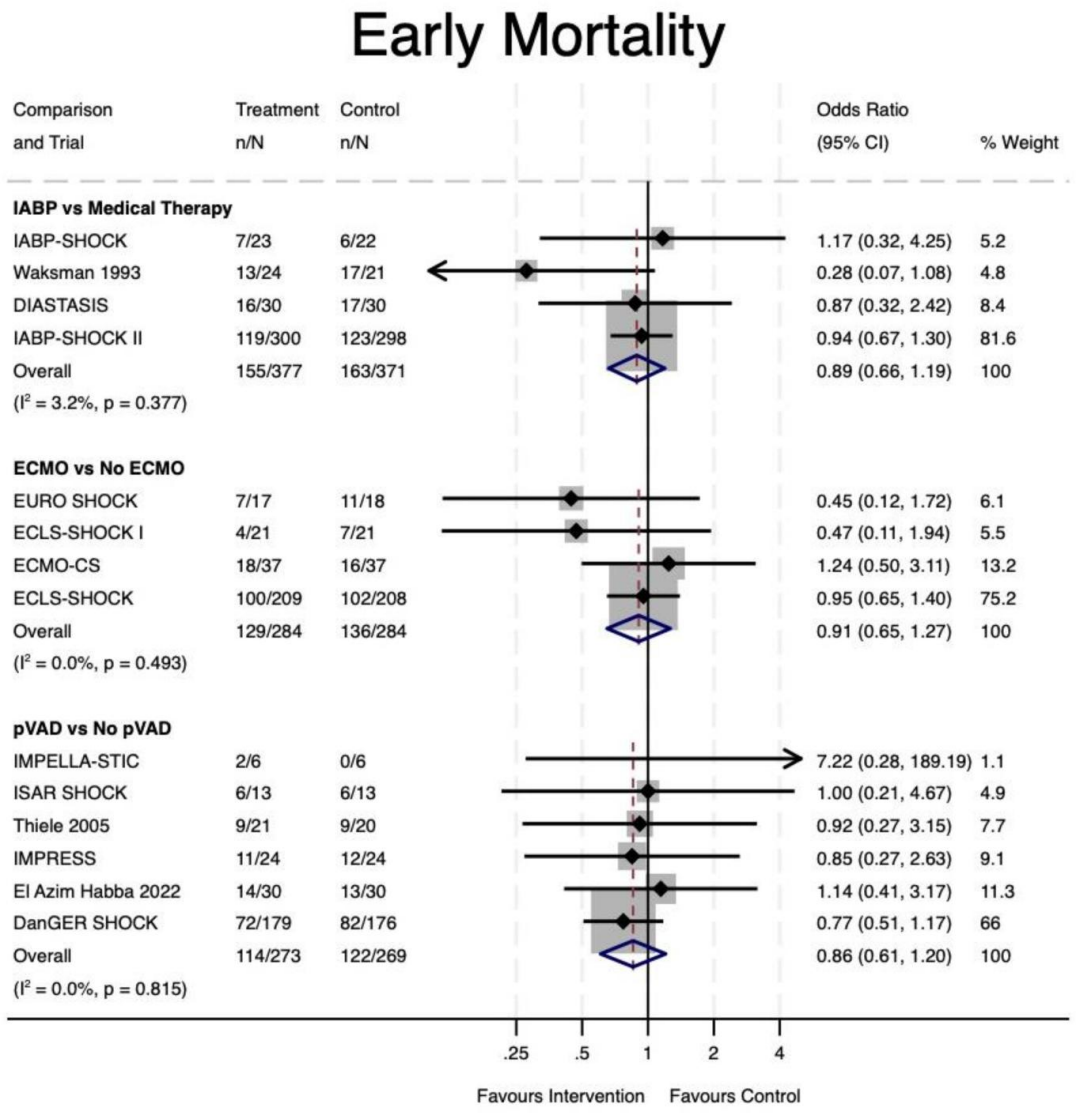
Early mortality (odds ratio). IABP, intra-aortic balloon pump; ECMO, extra-corporeal membrane oxygenation; pVAD, percutaneous ventricular assist device; CI, confidence interval.

In the pairwise meta-analyses, there was no statistically significant improvement in early mortality with any MCS device (*Figure 2*). IABP vs. medical therapy (OR 0.88, 95% CI 0.66–1.19, *P* = 0.43), ECMO vs. No ECMO (OR 0.91, 95% CI 0.65–1.27, *P* = 0.56), pVAD vs. No pVAD (OR 0.88, 95% CI 0.61–1.20, *P* = 0.37). There was no substantial statistical heterogeneity identified in any pairwise analysis (*I*^2^ < 3.3%, *P* > 0.37).

**Figure 3:**
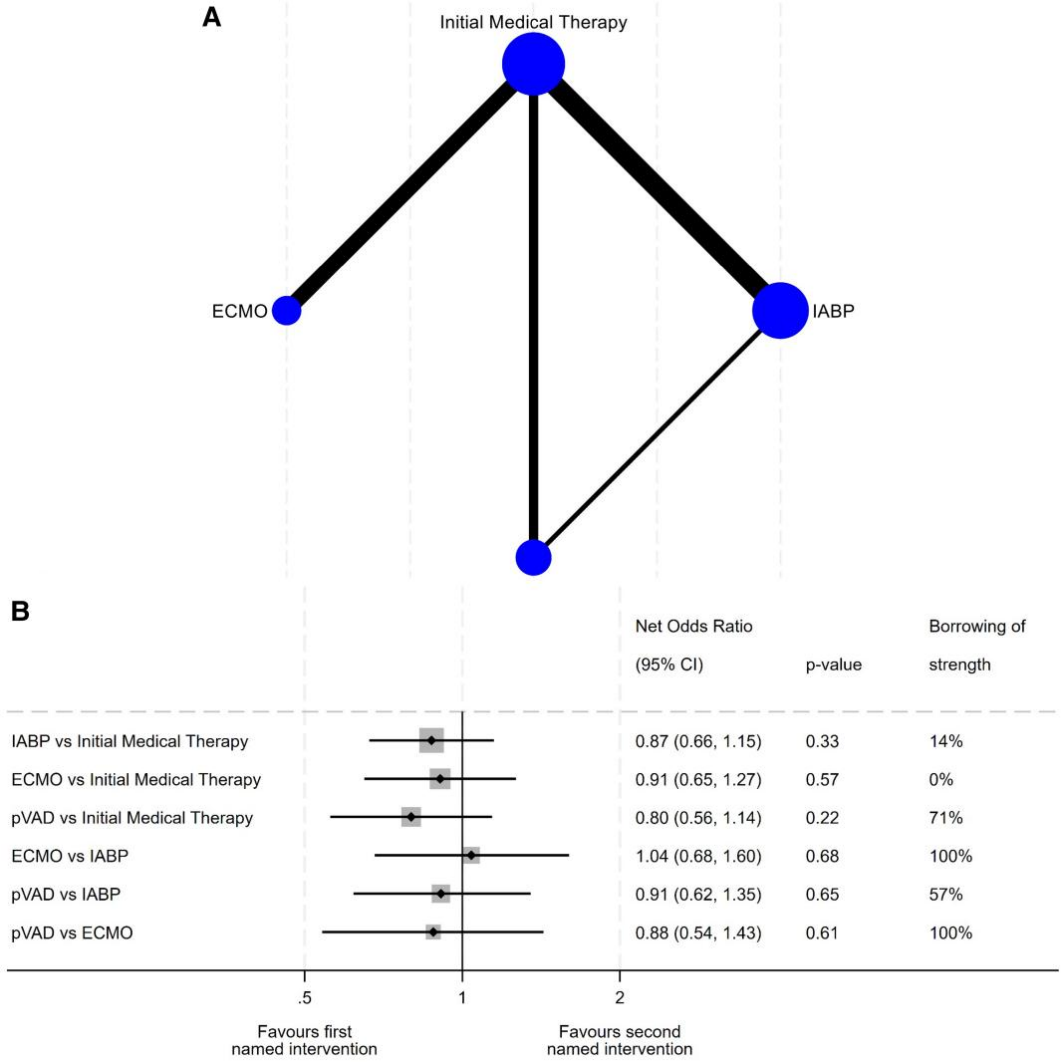
Network meta-analysis. Network map (*A*). Width of edges is proportional to the number of events in the comparison. Size of node is proportional to the number of trials that includes that intervention. Network meta-analysis forest plot (*B*). Each device compared to Initial Medical Therapy or alternative device with respect to effect on early mortality. IABP, intra-aortic balloon pump; ECMO, extra-corporeal membrane oxygenation; pVAD, percutaneous ventricular assist device; CI, confidence interval.

The network meta-analysis demonstrated similar results (*Figure 3*). There was no evidence of inconsistency within the network meta-analysis (*P*-value for inconsistency, *P* = 0.76).

All sensitivity analyses for early mortality gave consistent results for each comparison (see Supplementary material online, *Table S4*), and we did not identify variations of effects for early mortality in any of our subgroup analyses (see Supplementary material online, *Tables S5* and *S6*) indicating that these results appear robust to both methodological and clinical heterogeneity between the included trials. One-year mortality could not be included in the meta-analysis as data were only available for three studies. Pre-planned subgroup analysis by country was also not feasible due to the small number of trials conducted outside of the Europe (*n* = 4). The sensitivity analysis for the network meta-analysis excluding one trial (*n* = 12) that compared pVAD and IABP vs. IABP alone^12^ gave almost identical results.

### Safety

**Figure 4:**
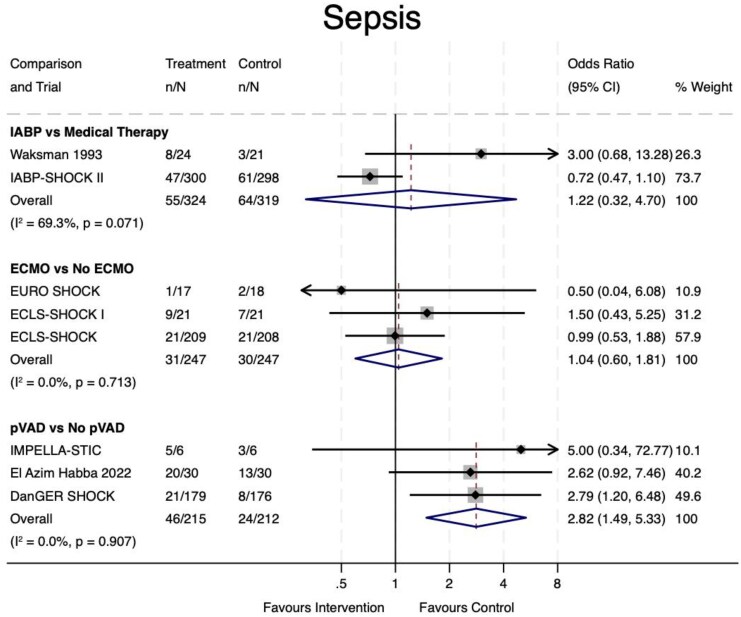
Sepsis. IABP, intra-aortic balloon pump; ECMO, extra-corporeal membrane oxygenation; pVAD, percutaneous ventricular assist device; CI, confidence interval.

Eight trials (86% of the overall sample) reported data on sepsis rates (*Figure 4*). There was a statistically significant increase in sepsis events with pVAD vs. No pVAD use (OR 2.82, 95% CI 1.49–5.33, *P* = 0.001), which was associated with low statistical heterogeneity (*I*^2^ = 0%, *P* = 0.907). The NNH was 9.

No such difference was found for VA-ECMO vs. No VA-ECMO (OR 1.40, 95% CI 0.60–1.81, *P* = 0.88) or IABP vs. medical therapy (OR 1.22, 95% CI 0.32–4.70, *P* = 0.77); although, the latter comparison was limited to only two trials with high heterogeneity (*I*^2^ = 69.3%, *P* = 0.071).

**Figure 5:**
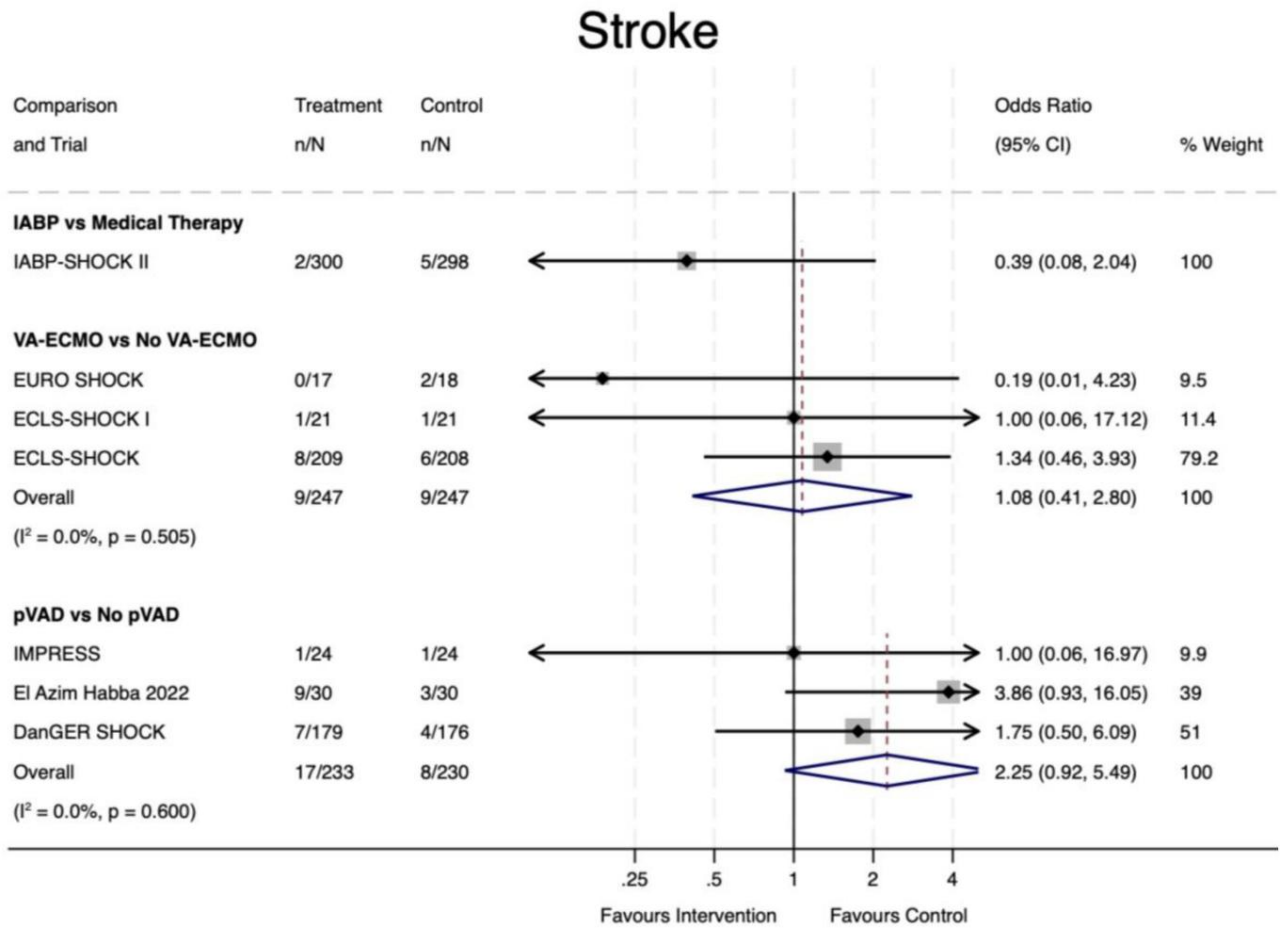
Stroke. IABP, intra-aortic balloon pump; ECMO, extra-corporeal membrane oxygenation; pVAD, percutaneous ventricular assist device; CI, confidence interval.

Data on stroke rates were available for a total of seven trials (84% of overall sample). There was no evidence of a difference in stroke events with IABP compared to initial medical therapy (OR 0.39, 95% CI 0.08–2.04, *P* = 0.27); however, data were only available for one trial, and 7/598 (0.01%) events reported overall (*Figure 5*). No difference in stroke rates was noted with VA-ECMO vs. No VA-ECMO (OR 1.08, 95% CI 0.41–2.80, *P* = 0.88). A trend of increased stroke was evident with pVAD use (OR 2.25, 95% CI 0.92–5.49, *P* = 0.07); however, this did not reach statistical significance. Statistical heterogeneity was low in both latter analyses (*I*^2^ = 0%, *P* > 0.5).

**Figure 6:**
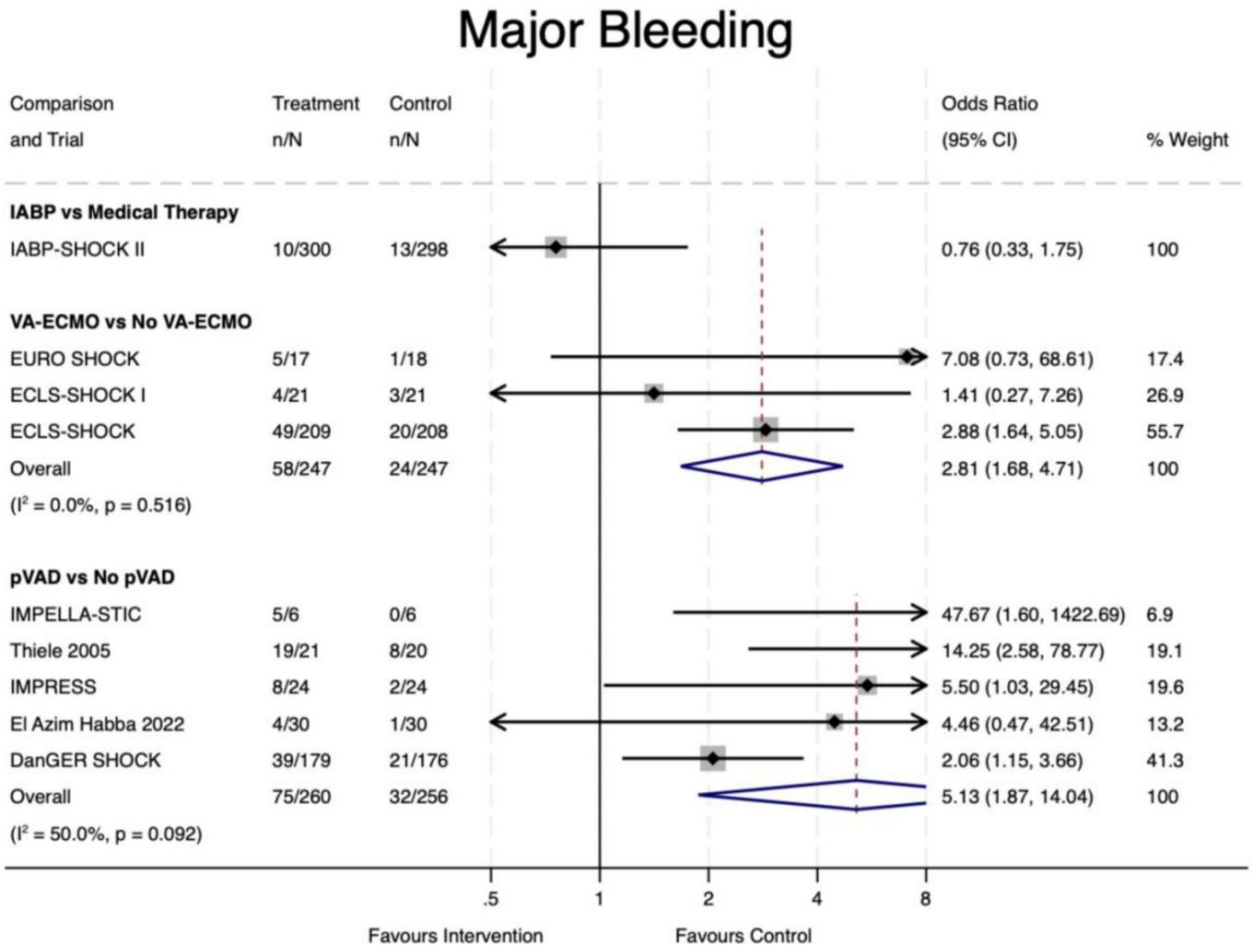
Major bleeding. IABP, intra-aortic balloon pump; ECMO, extra-corporeal membrane oxygenation; pVAD, percutaneous ventricular assist device; CI, confidence interval.

Nine trials (87% of the overall sample) provided data on major bleeding (*Figure 6*). Definitions for major bleeding are provided in Supplementary material online, *Table S7*. The sole IABP trial included in this analysis reported no significant increase in major bleeding with IABP compared to medical therapy (OR 0.76, 95% CI 0.33–1.75, *P* = 0.51). Bleeding was significantly increased with VA-ECMO (OR 2.81, 95% CI 1.68–4.71, *P* < 0.001) and pVAD (OR 5.13, 95% CI 1.87–14.04, *P* = 0.001). There was moderate statistical heterogeneity in the latter analysis (*I*^2^ = 50.0%, *P* = 0.092), which may be due to greater odds of bleeding for TandemHeart compared to Impella devices, although there was still strong statistical evidence of increased bleeding with Impella devices (see Supplementary material online, *Table S5*). The NNH was seven and six for VA-ECMO and pVAD, respectively.

Rates of urgent dialysis (three trials, OR 1.82, 95% CI 1.23–2.68, *P* = 0.003, NNH 7) and acute limb ischaemia (two trials, OR 6.97, 95% CI 1.79–27.12, *P* = 0.005, NNH 13) were both significantly increased with pVAD (see Supplementary material online, *Figures S1* and *S2*). There was no evidence of statistical heterogeneity for either meta-analysis. Data for these outcomes was available for 85% and 73% of the overall pVAD sample, respectively.

Limited IABP and VA-ECMO trials reported data on urgent renal replacement therapy (*n* = 3), limb ischaemia (*n* = 2), and average duration of hospital stay (*n* = 3). Furthermore, few trials (*n* = 5) reported data on major vascular complications, and there was significant heterogeneity in the definitions for those that did.

## Discussion

Our study found no significant early mortality benefit with any temporary MCS device compared to initial medical therapy and/or alternative MCS devices in adults with infarct-related cardiogenic shock planned for early revascularization. While a trend towards a modest, albeit statistically insignificant, treatment effect of MCS compared to initial medical therapy was observed for each device, the majority of trials conducted to date have been small and insufficiently powered to detect this effect. Furthermore, we observed an association of increased major safety events with MCS, particularly VA-ECMO and pVAD.

A recent individual participant data (IPD) meta-analysis of nine randomized trials (*n* = 1059) assessing VA-ECMO and pVAD in an AMI-CS population showed a similar trend towards modest benefit of early unselected MCS use compared to control on 6-month mortality (HR 0.87 [95% CI 0.74–1.03], *P* = 0.10).^27^ This IPD meta-analysis identified a clear benefit of MCS devices for those patients with ST-elevation myocardial infarction and low risk of hypoxic brain injury, concluding that ‘MCS should only be reserved for selected patients’. In our meta-analysis based on summary data, we did not have access to sufficient granularity from published trial reports to confirm or dispute this finding. However, our network meta-analysis, which borrows strength across comparisons to increase precision and has similarities to their main comparison of MCS devices vs. control, found similarly that there was no evidence that MCS devices provided a substantial treatment benefit on mortality on average. Similarities and differences between our study and the IPD meta-analysis by Thiele *et al*.^27^ is provided in Supplementary material online, *Table S8*.

Reporting of safety data were limited, which may due to numerous reasons. Safety outcomes were not pre-defined endpoints in many trials; particularly, the IABP trials where only IABP-SHOCK II appeared to have pre-specified safety endpoints.^13^ Secondly, the lack of a standardized endpoint framework for MCS trials may explain the heterogeneity in reporting of safety outcomes. This is highlighted by heterogeneity in definitions of major bleeding used across the trials (see Supplementary material online, *Table S4*). Nevertheless, a signal of potential harm was identified for pVAD devices, which were associated with significantly increased sepsis, major bleeding, dialysis, and limb ischaemia rates with a trend towards higher stroke rates. VA-ECMO was also associated with significantly more major bleeding. A similar hazard of increased major bleeding and vascular complications were reported with pVAD and VA-ECMO use compared to control in the recent IPD analysis.^27^

Differences in device characteristics may explain differences in safety hazards between IABP and other MCS.^2^ IABP insertion does not require large-bore cannulation unlike Impella and VA-ECMO, the latter requiring both arterial and venous access. This is likely to reduce the risk of sepsis, major bleeding and vascular complications with IABP’s. VA-ECMO introduces retrograde non-pulsatile aortic flow, which may be pro-thrombotic and increase the risk of vascular complications, stroke and limb ischaemia. Similarly, micro-axial flow pumps are associated with higher shear stress than counter-pulsation which may lead to haemolysis and thrombosis. Routine use of anti-coagulation with pVAD’s and VA-ECMO, unlike IABP, is also likely to confer a higher risk of stroke and bleeding. Finally, site and operator experience may play a role in improved safety with IABP’s, which are more established and more frequently used in clinical practice than other MCS.^7^ The influence of device familiarity on outcomes in MCS trials is highlighted by the DanGer shock trial,^15^ where significant heterogeneity was noted in the treatment effect based on the country of enrolment.

Overall, device-related complications may counterbalance any potential mortality benefit with MCS. Conversely, the observation of increased safety events in MCS arms could be a consequence of potential survival bias secondary to a mortality benefit with MCS. Increasing operator experience, device innovation and judicious patient selection may improve the safety of MCS in clinical practice.

### Intra-aortic balloon pump

These devices are the most popular temporary MCS devices in clinical practice for infarct-related shock, i.e. used in up to 40% of such cases in the Western world.^7^ The available clinical trial data does not support their frequent use in this setting as no trial met its primary endpoint. The majority of trials were small and even the larger and robustly designed IABP-SHOCK II trial^13^ reported 15% lower than expected mortality in its control arm, suggesting no trial was adequately powered to assess early mortality. There were a notably high proportion of patients in the control arm in IABP-SHOCK II who received IABP or another form of MCS which may have diluted any treatment effect, highlighting the challenges of conducting such trials.

Nevertheless, surrogate efficacy outcomes were similarly unsupportive of IABP efficacy. Trends in markers of end-organ dysfunction (lactate) and systemic inflammation (C-reactive protein) were similar between arms in IABP-SHOCK II. Similarly, there was no reduction in inotrope requirement or markers of congestion such as pulmonary capillary wedge pressure in IABP SHOCK I,^21^ or improvements in invasively measured haemodynamics such as cardiac output and systemic vascular resistance in the DIASTASIS study.^22^ The latter trial is likely at high risk-of-bias in assessing early mortality due to a lack of allocation concealment. Similarly, interpretation of Waksman *et al.*^3^ is limited by a flawed randomization process without allocation concealment and probable selection bias highlighted by an unusually high mortality rate (81%) in the control group. The overall rate of percutaneous revascularization was also low (38%) and heavily skewed in favour of the IABP arm (16:1), which is likely to have confounded results due to its proven efficacy in this setting.^5^

There was limited reporting of safety data in the IABP trials precluding meta-analyses or a descriptive comparison of pre-defined outcomes; however, the IABP-SHOCK II trial did not find any signal of increased sepsis, stroke, major bleeding, or vascular complications with IABP use compared to medical therapy. Despite similar definitions for cardiogenic shock (*Table 1*), mortality in the control arm was highly variable between trials (*Figure 2*), suggesting some clinical heterogeneity, which may limit interpretation of the results of this pairwise meta-analysis. Nevertheless, more randomized trial evidence is required to support the ongoing frequent use of IABP in AMI-CS in clinical practice. Two large Chinese trials are ongoing,^28,29^ with the former enrolling patients in the earlier stages of shock (SCAI-B). The recruitment target for these trials is 512 and 280 patients, respectively. Only one small unpublished IABP trial was identified.^30^ This trial was terminated pre-maturely due to insufficient recruitment (34 patients enrolled).

### Veno-arterial extra-corporeal membrane oxygenation

Similar to IABP, VA-ECMO has not demonstrated a signal of clinical or haemodynamic efficacy in AMI-CS trials to date, with no trial meeting its primary endpoint. ECLS-SHOCK I reported a numerically lower 30-day mortality with VA-ECMO; however, this was a small single-centre study with prognostically relevant imbalances in baseline characteristics, e.g. age and multi-vessel disease, favouring the intervention arm.^8^ The EURO-SHOCK investigators reported a similar trend towards reduced mortality with VA-ECMO; however, they recruited a highly selected cohort who had persistent signs of shock after PCI and did not have prolonged CPR.^23^ Despite being under-powered due to the impact of the COVID-19 pandemic, the high-screen failure rate (87%) in this trial limits the generalizability of any potential treatment effect to clinical practice.

The largest and most robust VA-ECMO trial to date, i.e. ECLS-SHOCK^16^ did not find any evidence of a beneficial treatment effect across its primary and secondary efficacy outcomes, or any subgroup interaction(s). This trial enrolled a significant proportion of patients with advanced shock (median lactate 6.8 mmol/L, 35% SCAI-E classification), who may have had irreversible multi-organ dysfunction at randomization. This was supported by similar times to haemodynamic stabilization and duration of catecholamine therapy in both groups; however, no signal of haemodynamic efficacy with VA-ECMO was evident in ECLS-SHOCK I despite enrolment of a lower-risk AMI-CS cohort.^8^ Furthermore, an IPD meta-analysis of recent MCS trials found that subgroups with potentially more advanced haemo-metabolic compromise may derive a greater 6-month mortality benefit most from MCS compared to control.^27^ This included patients with higher lactate, lower SBP and those with unsuccessful PCI.

Notably, rates of left ventricular unloading with a concomitant device was uniformly low in VA-ECMO trials despite observational evidence of a potential mortality benefit with this strategy, which aims to counteract the deleterious effect of VA-ECMO on ventricular afterload.^31^ The recent IPD meta-analysis by Thiele *et al*. reported no statistically significant reduction in 6-month mortality in patients receiving ventricular unloading devices compared to control (HR 0.80 [95% CI 0.62–1.02]; *P* = 0·075).^27^ However, the control arm of most of these trials included IABP, another unloading device. The ongoing ANCHOR trial, which is planning to enrol 400 patients, is investigating whether a systematic left ventricular unloading strategy using an IABP may improve early mortality compared to medical therapy alone.^32^

Our study found higher rates of major bleeding with VA-ECMO (NNH of 7). Reporting of other safety outcomes was poor in VA-ECMO trials; however, higher rates of major vascular complications (RR 2.86, 95% CI 1.31–6.25) compared to medical therapy were noted in ECLS-SHOCK,^16^ while numerically higher rates were noted in the under-powered ECMO-CS^9^ and EURO-SHOCK^23^ trials. These complications are unsurprising given the large-bore cannulation required for vascular access to use this device. Furthermore, significantly longer intensive care unit stay and duration of ventilation was reported for VA-ECMO survivors compared to control survivors in EURO-SHOCK. These findings suggest there are significant risks associated with VA-ECMO use, which may partially explain the lack of mortality benefit over medical therapy in trials to date. Future studies should aim to identify predictors of major complications to guide optimal patient selection in future VA-ECMO trials.

### Percutaneous ventricular assist devices

Only one single small trial has been evaluated the efficacy of TandemHeart in AMI-CS.^25^ This study showed some evidence of early haemodynamic efficacy; however, use of the device was associated with significantly higher bleeding, vascular complications, and procedural time compared to IABP. The IMPELLA device is the most well-studied pVAD. Unlike the IABP and VA-ECMO trials, these trials primarily used IABP controls except for DanGer SHOCK.^15^ Cardiogenic shock definitions were also more heterogeneous in this group (*Table 1*) Most were small phase II trials designed to assess haemodynamic efficacy.^10-12^ Of these, only ISAR-SHOCK met its primary endpoint of change in cardiac index at 30 min; however, this hyper-acute benefit was neither sustained after 24 h nor supported by improvements in other haemodynamic parameters or clinical endpoints, limiting their clinical relevance. While IMPELLA-CP was the most commonly used IMPELLA device across trials, our subgroup analysis did not observe any significant difference in efficacy and safety outcomes with this device compared to other pVAD’s.

To date, DanGer SHOCK is the only MCS trial in AMI-CS to meet its primary clinical endpoint, i.e. 180-day all-cause mortality. The investigators chose to exclude patients with out-of-hospital cardiac arrest or those in a persistent comatose state on arrival to hospital, which were strong predictors of neurological death in IMPRESS.^11^ Many AMI-CS patients present in this way, which limits the generalizability of the DanGer SHOCK trial results due to its high screen failure rate (70%) compared to other trials, such as IMPRESS (36%) and ECLS-SHOCK (52%). Furthermore, the statistical significance of the primary endpoint was fragile (*P* = 0.04, upper limit CI = 0.99), highlighted by a paradoxically more conservative estimate in the as-treated population, which did not show a statistically significant mortality benefit. There was also no 30-day mortality difference between groups and significant heterogeneity was noted in the treatment effect based on the country of enrolment (the study was limited to three European sites). Nevertheless, DanGer SHOCK has highlighted that MCS may benefit carefully selected shock patients at reduced risk of hypoxic brain injury. This hypothesis was further supported by data from the recent IPD meta-analysis of MCS trials in infarct-related shock which reported that the subgroup of patients who had <10 min or no cardiopulmonary resuscitation (*n* = 589) appeared to benefit most from MCS (HR 0.77, [95% CI 0.61–0.97], *P* = 0.024).^27^

Our study also highlights some safety concerns associated with pVAD use: we note significantly higher rates of sepsis, major bleeding, urgent dialysis, and limb ischaemia. There was also a trend towards higher stroke rates. Therefore, despite the encouraging results of DanGer SHOCK, its limitations along with these safety concerns with pVAD use highlight the need for more randomized trial data. While it is unfortunate that RECOVERY IV^33^ was recently terminated early after recruitment of five patients based on the results of DanGer SHOCK, results of the ongoing ULYSS trial in France, which plans to enrol 204 patients may be important.^34^ One other small IMPELLA trial was terminated early due to insufficient recruitment (19 patients enrolled).^35^

### Limitations

The majority of trials included in this analysis were small and baseline characteristics were difficult to compare due to variable reporting. Therefore, while statistical heterogeneity was low overall in most analyses, clinical heterogeneity was difficult to assess. The timing of MCS insertion may have varied in the included studies and shock severity was difficult to compare between the studies. Only a small number of trials were available per comparison. In addition, due to heterogeneous and/or limited reporting of outcomes by participant-level factors in the included trials, we were limited to trial-level subgroup analyses that may not have fully captured differences in outcomes due clinical heterogeneity. However, we did not identify substantial statistical heterogeneity for our primary outcome of early mortality for any of our three comparisons. There was limited reporting of safety data (including outcome definitions) and long-term efficacy outcomes, and insufficient data to undertake the pre-planned subgroup analysis. While Impella CP was the most commonly used pVAD, 26% of patients randomized to intervention across the pVAD trials received an alternative micro-axial flow pump, which may have influenced the evaluation of safety outcomes.

## Conclusion

The current randomized trial evidence base does not provide robust evidence of an early mortality benefit with any MCS strategy currently used in clinical practice. However, these devices may confer a treatment benefit, particularly in selected subgroups at lower risk of hypoxic brain injury, as highlighted by the findings of DanGer shock and a recent IPD meta-analysis.^15,27^ Despite limited reporting of safety data in trials to date, there is some evidence of serious risks associated with VA-ECMO and pVAD use in particular. However, contextual factors including operator experience, access techniques, and device selection may have significantly influenced safety and efficacy outcomes. Future trials should explore whether these devices may be beneficial in the earlier stages of shock when the risk of irreversible multi-organ dysfunction may be lower. Standardized definitions should be incorporated for relevant safety outcomes, with transparent reporting of such outcomes.

## Supplementary Material

oeaf091_Supplementary_Data

## Data Availability

The data underlying this article will be shared on reasonable request by the corresponding author.

## Lead author biography

Dr Zaran Butt, MB BCh BAO, MRCPI, MSc, is a cardiology specialist registrar currently training in Dublin, Ireland. He completed his medical degree at University College Dublin and is undertaking full-time clinical training on the higher specialist training programme in Ireland, with a particular interest in interventional cardiology. He also has a keen interest in clinical research, in particular clinical trials. He was awarded an MSc in clinical trials with distinction from University College London in 2024.
