# Supplementary material for: Mechanical circulatory support for infarct-related cardiogenic shock: a systematic review, pairwise and network meta-analysis
Source: Eur Heart J Open. 2025 Jul 29;5(4):oeaf091. doi: 10.1093/ehjopen/oeaf091 (PMC12342370; doi:10.1093/ehjopen/oeaf091)
Supplement: oeaf091_Supplementary_Data [file oeaf091_supplementary_data.docx]

Table of Contents

[Supplementary Table 1. PRISMA Checklist 2](#_Toc195897805)

[Supplementary Table 2. PICOS framework 6](#_Toc195897806)

[Supplementary Table 3. Detailed risk of bias assessment at study level with individual domains for the outcome of early mortality. 8](#_Toc195897807)

[Supplementary Table 4. Sensitivity analyses for Early mortality 9](#_Toc195897808)

[Supplementary Table 5: Analyses by device type for pVAD vs no pVAD comparison 10](#_Toc195897809)

[Supplementary Table 6: Trial-level subgroup analysis by multi/single-centre trial 11](#_Toc195897810)

[Supplementary Table 7. Major bleeding definitions 14](#_Toc195897811)

[Supplementary Table 8. Comparison of our pairwise and network meta-analysis with a recent IPD meta-analysis by Thiele et al. 17](#_Toc195897812)

[Search Strategies 18](#_Toc195897813)

[Medline : 18](#_Toc195897814)

[CENTRAL (Cochrane library) : 24](#_Toc195897815)

[Embase : 26](#_Toc195897816)

[Scopus : 30](#_Toc195897817)

[Web of Science : 31](#_Toc195897818)

[Supplementary Figure 1. Urgent dialysis (pVAD) 35](#_Toc195897819)

[Supplementary Figure 2. Limb Ischaemia (pVAD) 36](#_Toc195897820)

# Supplementary Table 1. PRISMA Checklist

| **Section/Topic** | **Item #** | **Checklist Item** | **Reported on Page #** |
| --- | --- | --- | --- |
| **TITLE** |  |  |  |
| Title | 1 | Identify the report as a systematic review *incorporating a network meta-analysis (or related form of meta-analysis).* | 1 |
|  |  |  |  |
| **ABSTRACT** |  |  |  |
| Structured summary | 2 | Provide a structured summary including, as applicable:  **Background:** main objectives  **Methods:** data sources; study eligibility criteria, participants, and interventions; study appraisal; and *synthesis methods, such as network meta-analysis.*  **Results:** number of studies and participants identified; summary estimates with corresponding confidence/credible intervals; *treatment rankings may also be discussed. Authors may choose to summarize pairwise comparisons against a chosen treatment included in their analyses for brevity.*  **Discussion/Conclusions:** limitations; conclusions and implications of findings.  **Other:** primary source of funding; systematic review registration number with registry name. | 2-3 |
|  |  |  |  |
| **INTRODUCTION** |  |  |  |
| Rationale | 3 | Describe the rationale for the review in the context of what is already known*, including mention of why a network meta-analysis has been conducted.* | 4-5 |
| Objectives | 4 | Provide an explicit statement of questions being addressed, with reference to participants, interventions, comparisons, outcomes, and study design (PICOS). | 5 |
|  |  |  |  |
| **METHODS** |  |  |  |
| Protocol and registration | 5 | Indicate whether a review protocol exists and if and where it can be accessed (e.g., Web address); and, if available, provide registration information, including registration number. | 6 |
| Eligibility criteria | 6 | Specify study characteristics (e.g., PICOS, length of follow-up) and report characteristics (e.g., years considered, language, publication status) used as criteria for eligibility, giving rationale. *Clearly describe eligible treatments included in the treatment network, and note whether any have been clustered or merged into the same node (with justification).* | 6 |
| Information sources | 7 | Describe all information sources (e.g., databases with dates of coverage, contact with study authors to identify additional studies) in the search and date last searched. | 6 |
| Search | 8 | Present full electronic search strategy for at least one database, including any limits used, such that it could be repeated. | Appendix |
| Study selection | 9 | State the process for selecting studies (i.e., screening, eligibility, included in systematic review, and, if applicable, included in the meta-analysis). | 6-7 |
| Data collection process | 10 | Describe method of data extraction from reports (e.g., piloted forms, independently, in duplicate) and any processes for obtaining and confirming data from investigators. | 6-7 |
| Data items | 11 | List and define all variables for which data were sought (e.g., PICOS, funding sources) and any assumptions and simplifications made. | 6-7 |
| **Geometry of the network** | **S1** | Describe methods used to explore the geometry of the treatment network under study and potential biases related to it. This should include how the evidence base has been graphically summarized for presentation, and what characteristics were compiled and used to describe the evidence base to readers. | 8 |
| Risk of bias within individual studies | 12 | Describe methods used for assessing risk of bias of individual studies (including specification of whether this was done at the study or outcome level), and how this information is to be used in any data synthesis. | 7 |
| Summary measures | 13 | State the principal summary measures (e.g., risk ratio, difference in means). *Also describe the use of additional summary measures assessed, such as treatment rankings and surface under the cumulative ranking curve (SUCRA) values, as well as modified approaches used to present summary findings from meta-analyses.* | 7-8 |
| Planned methods of analysis | 14 | Describe the methods of handling data and combining results of studies for each network meta-analysis. This should include, but not be limited to:   - *Handling of multi-arm trials;* - *Selection of variance structure;* - *Selection of prior distributions in Bayesian analyses; and* - *Assessment of model fit.* | 8 |
| **Assessment of Inconsistency** | **S2** | Describe the statistical methods used to evaluate the agreement of direct and indirect evidence in the treatment network(s) studied. Describe efforts taken to address its presence when found. | 8 |
| Risk of bias across studies | 15 | Specify any assessment of risk of bias that may affect the cumulative evidence (e.g., publication bias, selective reporting within studies). | 7 |
| Additional analyses | 16 | Describe methods of additional analyses if done, indicating which were pre-specified. This may include, but not be limited to, the following:   - Sensitivity or subgroup analyses; - Meta-regression analyses; - *Alternative formulations of the treatment network; and* - *Use of alternative prior distributions for Bayesian analyses (if applicable).* | 8 |
|  |  |  |  |
| **RESULTS†** |  |  |  |
| Study selection | 17 | Give numbers of studies screened, assessed for eligibility, and included in the review, with reasons for exclusions at each stage, ideally with a flow diagram. | Figure 1. |
| **Presentation of network structure** | **S3** | Provide a network graph of the included studies to enable visualization of the geometry of the treatment network. | Figure 3A |
| **Summary of network geometry** | **S4** | Provide a brief overview of characteristics of the treatment network. This may include commentary on the abundance of trials and randomized patients for the different interventions and pairwise comparisons in the network, gaps of evidence in the treatment network, and potential biases reflected by the network structure. | 9-10, Figure 3A |
| Study characteristics | 18 | For each study, present characteristics for which data were extracted (e.g., study size, PICOS, follow-up period) and provide the citations. | 9-10, Table 1. |
| Risk of bias within studies | 19 | Present data on risk of bias of each study and, if available, any outcome level assessment. | 9, Supp Table 3. |
| Results of individual studies | 20 | For all outcomes considered (benefits or harms), present, for each study: 1) simple summary data for each intervention group, and 2) effect estimates and confidence intervals. *Modified approaches may be needed to deal with information from larger networks.* | 9-12, Figure 2, Figure 4-8 |
| Synthesis of results | 21 | Present results of each meta-analysis done, including confidence/credible intervals. *In larger networks, authors may focus on comparisons versus a particular comparator (e.g. placebo or standard care), with full findings presented in an appendix. League tables and forest plots may be considered to summarize pairwise comparisons.* If additional summary measures were explored (such as treatment rankings), these should also be presented. | 9-12, Figure 2, Figure 4-8 |
| **Exploration for inconsistency** | **S5** | Describe results from investigations of inconsistency. This may include such information as measures of model fit to compare consistency and inconsistency models, *P* values from statistical tests, or summary of inconsistency estimates from different parts of the treatment network. | 10 |
| Risk of bias across studies | 22 | Present results of any assessment of risk of bias across studies for the evidence base being studied. | Table 1. |
| Results of additional analyses | 23 | Give results of additional analyses, if done (e.g., sensitivity or subgroup analyses, meta-regression analyses*, alternative network geometries studied, alternative choice of prior distributions for Bayesian analyses,* and so forth). | 10 |
|  |  |  |  |
| **DISCUSSION** |  |  |  |
| Summary of evidence | 24 | Summarize the main findings, including the strength of evidence for each main outcome; consider their relevance to key groups (e.g., healthcare providers, users, and policy-makers). | 13-18 |
| Limitations | 25 | Discuss limitations at study and outcome level (e.g., risk of bias), and at review level (e.g., incomplete retrieval of identified research, reporting bias). *Comment on the validity of the assumptions, such as transitivity and consistency. Comment on any concerns regarding network geometry (e.g., avoidance of certain comparisons).* | 19 |
| Conclusions | 26 | Provide a general interpretation of the results in the context of other evidence, and implications for future research. | 19 |
|  |  |  |  |
| **FUNDING** |  |  |  |
| Funding | 27 | Describe sources of funding for the systematic review and other support (e.g., supply of data); role of funders for the systematic review. This should also include information regarding whether funding has been received from manufacturers of treatments in the network and/or whether some of the authors are content experts with professional conflicts of interest that could affect use of treatments in the network. | 30 |

**Supplementary Table 1.** PRISMA Checklist for Systematic Reviews and Network Meta-analyses (2020)

| **Population** | Adults (>18 years old) with infarct-related cardiogenic shock planned for early revascularization (primary percutaneous coronary intervention or coronary artery bypass grafting) |
| --- | --- |
| **Intervention** | Temporary mechanical circulatory support (any modality) |
| **Control** | Medical Therapy (inotropes) and/or other Mechanical Circulatory Support |
| **Outcome** | Early mortality (30-day or in-hospital all-cause mortality) |
| **Study Design** | Randomized-controlled trials only |

Supplementary Table 2. PICOS framework for the study

|  | Randomization process | Deviation from intended intervention | Missing outcome data | Measurement of outcome | Selection of reported result | Overall |
| --- | --- | --- | --- | --- | --- | --- |
| Intra-aortic balloon pump: | | | | | | |
| Prondzinsky *et al.* 2010 | Low | Low | Low | Low | Low | Low |
| Sharma *et al.* 2022 | High | Some Concerns | Low | Low | Low | High |
| Thiele *et al.* 2012 | Low | Some Concerns | Low | Low | Low | Some Concerns |
| Waksman *et al.* 1993 | High | Low | Low | Low | Low | High |
| Veno-arterial extracorporeal membrane oxygenation: | | | | | | |
| Ostadal *et al.* 2023 | Low | High | Low | Low | Low | High |
| Banning *et al.* 2023 | Some Concerns | Some Concerns | Low | Low | Low | Some Concerns |
| Brunner *et al.* 2019 | High | Some Concerns | Low | Low | Some Concerns | High |
| Thiele *et al.* 2023 | Low | Some Concerns | Low | Low | Low | Some Concerns |
| Percutaneous ventricular assist device: | | | | | | |
| Bonnefoy-Cudraz *et al.* 2014 | Some Concerns | Some Concerns | Low | Low | Low | Some Concerns |
| Moller *et al.* 2024 | Low | Some Concerns | Low | Low | Low | Some Concerns |
| Seyfarth *et al.* 2008 | Some Concerns | Some Concerns | Low | Low | Some Concerns | Some Concerns |
| Ouweneel *et al.* 2017 | Low | Some Concerns | Low | Low | Low | Some Concerns |
| El Azim Habba *et al.* 2022 | High | High | Low | Low | Low | High |
| Thiele *et al.* 2005 | Low | Low | Low | Low | Low | Low |

**Supplementary Table 3.** Detailed risk of bias assessment at study level with individual domains for the outcome of early mortality.

**Supplementary Table 3.** Detailed risk of bias assessment at study level with individual domains for the outcome of early mortality.

| **Analysis** | **IABP vs Medical Therapy** | | **ECMO vs No ECMO** | | **pVAD vs No pVAD** | |
| --- | --- | --- | --- | --- | --- | --- |
|  | **Trials/Patients** | **Estimate (95% CI)** | **Trials/Patients** | **Estimate (95% CI)** | **Trials/Patients** | **Estimate (95% CI)** |
| Main analysis (Figure 2) | 4/748 | 0.89 (0.66, 1.19) | 4/568 | 0.91 (0.65, 1.27) | 6/542 | 0.86 (0.61, 1.20) |
| Sensitivity analysis 1: Relative risk as effect measure | 4/748 | 0.91 (0.77, 1.08) | 4/568 | 0.96 (0.80, 1.14) | 6/542 | 0.91 (0.75, 1.11) |
| Sensitivity analysis 2: Excluding studies at High Risk of Bias | 2/643 | 0.95 (0.69, 1.30) | 2/452 | 0.87 (0.53, 1.42) | 5/482 | 0.82 (0.57, 1.18) |
| Sensitivity analysis 3: Limited to studies at Low Risk of Bias | 1/45 | 1.17 (0.32, 4.25) | 0/0 | - | 1/41 | 0.92 (0.27, 3.15) |
| Sensitivity analysis 4: Excluding studies with >15% crossover rate* | 4/748 | 0.89 (0.66, 1.19) | 2/452 | 0.87 (0.53, 1.42) | 5/482 | 0.82 (0.57, 1.18) |
| Sensitivity analysis 4: Limited to studies with 0% crossover rate* | 2/105 | 0.54 (0.18, 1.63) | 0/0 | - | 3/79 | 1.12 (0.44, 2.81) |

# Supplementary Table 4. Sensitivity analyses for Early mortality

All meta-analyses are performed using inverse-variance random-effects models [restricted maximum likelihood (REML) estimator of Tau^2^], and (unless stated) use odds ratios as the effect measure.

*Note that one study (ECLS-SHOCK I) from the ECMO vs No ECMO comparison is excluded from these analyses as crossover rate was not reported.

*CI* Confidence Interval, *IABP* Intra-aortic balloon pump, *ECMO* Extra-corporeal membrane oxygenation, *pVAD* Percutaneous ventricular assist device

# Supplementary Table 5: Analyses by device type for pVAD vs no pVAD comparison

| **Outcome** | **Device type trial-level subgroup analysis** | | | | | **Limited to Impella CP devices only in the intervention arm** | |
| --- | --- | --- | --- | --- | --- | --- | --- |
|  | **Impella (any) vs No Impella** | | **TandemHeart vs No TandemHeart** | | **p-value*** | **Impella CP vs No Impella CP** | |
|  | **Trials/Patients** | **OR (95% CI)** | **Trials/Patients** | **OR (95% CI)** |  | **Trials/Patients** | **OR (95% CI)** |
| Early Mortality | 5/501 | 0.85 (0.60, 1.21) | 1/41 | 0.92 (0.27, 3.15) | 0.91 | 3/463 | 0.82 (0.57, 1.18) |
| Sepsis | 3/427 | 2.82 (1.49, 5.33) | 0/0 | - | - | 2/415 | 2.72 (1.41, 5.25) |
| Stroke | 3/463 | 2.25 (0.92, 5.49) | 0/0 | - | - | 3/463 | 2.25 (0.92, 5.49) |
| Major Bleeding | 4/475 | 2.54 (1.51, 4.30) | 1/41 | 14.25 (2.58, 78.77) | 0.06 | 3/463 | 2.37 (1.39, 4.03) |

All meta-analyses are performed using inverse-variance random-effects models [restricted maximum likelihood (REML) estimator of Tau^2^], and use odds ratios as the effect measure.

*Each p-value is from a Wald test of subgroup differences between the Impella (any) vs No Impella subgroup estimate and the TandemHeart vs No TandemHeart estimate. Small p-values suggest that there is evidence against the null hypothesis that the two subgroup estimates are the same, i.e. that the efficacy/safety varies based on type of pVAD. Larger p-values suggest that there is no evidence that the efficacy/safety varies by pVAD type.

*OR* Odds Ratio, *CI* Confidence Interval, *pVAD* Percutaneous ventricular assist device

| **Comparison** | **IABP vs Medical Therapy** | | | | |
| --- | --- | --- | --- | --- | --- |
| **Outcome** | **Single-centre** | | **Multi-centre** | | **p-value*** |
|  | **Trials/Patients** | **OR (95% CI)** | **Trials/Patients** | **OR (95% CI)** |  |
| Early Mortality | 3/150 | 0.71 (0.35, 1.40) | 1/598 | 0.94 (0.67, 1.30) | 0.47 |
| Sepsis | 1/45 | 3.00 (0.68, 13.28) | 1/598 | 0.72 (0.47, 1.10) | 0.07 |
| Stroke | 0/0 | - | 1/598 | 0.39 (0.08, 2.04) | - |
| Major Bleeding | 0/0 | - | 1/598 | 0.76 (0.33, 1.75) | - |
|  | | | | | |
| **Comparison** | **ECMO vs No ECMO** | | | | |
| **Outcome** | **Single-centre** | | **Multi-centre** | | **p-value*** |
|  | **Trials/Patients** | **OR (95% CI)** | **Trials/Patients** | **OR (95% CI)** |  |
| Early Mortality | 1/42 | 0.47 (0.11, 1.94) | 3/526 | 0.94 (0.67, 1.33) | 0.35 |
| Sepsis | 1/42 | 1.50 (0.43, 5.25) | 2/452 | 0.95 (0.51, 1.77) | 0.53 |
| Stroke | 1/42 | 1.00 (0.06, 17.12) | 2/452 | 1.09 (0.39, 3.00) | 0.51 |
| Major Bleeding | 1/42 | 1.41 (0.27, 7.26) | 2/452 | 3.03 (1.76, 5.23) | 0.52 |
|  | | | | | |
| **Comparison** | **pVAD vs No pVAD** | | | | |
| **Outcome** | **Single-centre** | | **Multi-centre** | | **p-value*** |
|  | **Trials/Patients** | **OR (95% CI)** | **Trials/Patients** | **OR (95% CI)** |  |
| Early Mortality | 2/101 | 1.05 (0.48, 2.29) | 4/441 | 0.82 (0.56, 1.19) | 0.56 |
| Sepsis | 1/60 | 2.62 (0.92, 7.46) | 2/367 | 2.94 (1.32, 6.57) | 0.91 |
| Stroke | 1/60 | 3.86 (0.93, 16.05) | 2/403 | 1.60 (0.51, 5.00) | 0.60 |
| Major Bleeding | 2/101 | 9.33 (2.39, 36.41) | 3/415 | 2.46 (1.44, 4.22) | 0.09 |

# Supplementary Table 6: Trial-level subgroup analysis by multi/single-centre trial

All meta-analyses are performed using inverse-variance random-effects models [restricted maximum likelihood (REML) estimator of Tau^2^], and use odds ratios as the effect measure.

*Each p-value is from a Wald test of subgroup differences between the single-centre and multi-centre subgroup estimates. Small p-values suggest that there is evidence against the null hypothesis that the two subgroup estimates are the same, i.e. that the efficacy/safety varies based on whether trials were single-centre or multi-centre. Larger p-values suggest that there is no evidence that the efficacy/safety varies by centre.
*OR* odds ratio, *CI* confidence interval, *IABP* Intra-aortic balloon pump, *ECMO* Extra-corporeal membrane oxygenation, *pVAD* Percutaneous ventricular assist device

| Trial | Major Bleeding Definition |
| --- | --- |
| Intra-aortic balloon pump: |  |
| Prondzinsky *et al.* 2010 | N/A |
| Sharma *et al.* 2022 | N/A |
| Thiele *et al.* 2012 | Intracerebral bleed or bleed resulting in substantial hemodynamic compromise requiring treatment |
| Waksman *et al.* 1993 | N/A |
| VA-ECMO: |  |
| Ostadal *et al.* 2023 | N/A |
| Banning *et al.* 2023 | BARC 3-5 bleeding |
| Brunner *et al.* 2019 | N/A |
| Thiele *et al.* 2023 | BARC 3-5 bleeding |
| pVAD: |  |
| Bonnefoy-Cudraz *et al.* 2014 | Bleeding requiring any transfusion |
| Moller *et al.* 2024 | Intracerebral bleed or bleed resulting in substantial hemodynamic compromise requiring treatment or any bleed requiring transfusion |
| Seyfarth *et al.* 2008 | N/A |
| Ouweneel *et al.* 2017 | Bleeding with Hb drop >5g/dL or requiring >/= 2 units RCC or requiring surgical control |
| El Azim Habba *et al.* 2022 | Bleeding requiring any transfusion |
| Thiele *et al.* 2005  Supplementary Table 7. Major bleeding definitions for each trial  *BARC* Bleeding Academic Research Consortium, *VA-ECMO* Extra-corporeal membrane oxygenation, *pVAD* Percutaneous  ventricular assist device, *N/A* Not Available | Bleeding requiring any transfusion |

| **Characteristic** | **Our study** | **IPD meta-analysis** |
| --- | --- | --- |
| Study registration | PROSPERO (CRD42024546141) | PROSPERO (CRD42024504295) |
| Eligibility criteria | We included all randomized-controlled trials comparing use of active MCS (including IABP) versus control (including use of alternative MCS) in patients with AMI-CS planned for early revascularization. | The IPD analysis included all randomized-controlled trials comparing early routine use of active MCS (excluding IABP) versus best medical therapy (including use of IABP) in patients with AMI-CS undergoing revascularization without ongoing cardio-pulmonary resuscitation. |
| Overall Sample | 1858 patients | 1059 patients |
| Outcomes | We had one efficacy outcome of early mortality, that was either in-hospital or 30-day mortality.  We had several safety outcomes, including stroke, sepsis, major bleeding, urgent dialysis and limb ischaemia. | The IPD meta-analysis had similar outcomes, with a primary outcome of 6-month all-cause mortality and secondary safety outcomes that included stroke, sepsis, moderate/severe bleeding and peripheral ischaemic  vascular complications and renal replacement therapy.  There were additional outcomes including 30-day mortality, days of intensive care treatment and total duration of hospital stay. |
| Treatment comparisons | We split trials into three treatment comparisons to limit clinical heterogeneity from different MCS devices. We then used network meta-analysis to borrow strength across comparisons through using direct and indirect information, ensuring that the network meta-analysis consistency assumption was not violated, to provide precise estimates of treatment effect for our primary outcome of early mortality. | The IPD meta-analysis kept all trials together in one MCS device vs control comparison as their primary approach and then split into sub-comparisons as secondary analyses. |
| Assessing risk of bias | Cochrane Risk of Bias 2 tool for our primary outcome of early mortality | Cochrane Risk of Bias 2 tool for our primary outcome of 6-month mortality |
| Exploring clinical heterogeneity | We chose not to attempt to explore participant-level subgroup analyses due to the heterogeneity of reporting from the included trials. Any analyses that we could have performed would have had the potential to be impacted by aggregation bias.  Therefore, we focused on trial-level factors to explore in subgroup analyses or restricted trials in sensitivity analyses to test the robustness of our assumptions, all indicating the robustness of our primary finding of no clear benefit of any of the interventions on early mortality. | With IPD, participant-level subgroup analyses are possible and additional analyses, restricting trials to particular populations of patients to limit clinical heterogeneity. |
| Statistical approach to meta-analysis | We chose to fit inverse variance random-effects models using the recommended restricted likelihood estimator of the heterogeneity variance (tau^2^) to reflect the clinical heterogeneity between the studies in our pooled estimates. | One-stage fixed-effects (stratified) models, after having tested for the presence of statistical heterogeneity. These models increase the precision in the pooled estimate, but may not fully capture the required variability if there is some statistical heterogeneity present (e.g. due to clinical heterogeneity between the trials) |
| Efficacy findings | No MCS device showed a significant effect on early mortality versus initial medical therapy {IABP (OR 0.87, 95% CI 0.66 – 1.15), VA-ECMO (OR 0.91, 95% CI 0.65 – 1.27), pVAD (OR 0.80, 95% CI 0.56 to 1.14), P (inconsistency) = 0.76}. | No significant benefit of early MCS use on 6-month mortality was noted (HR 0.87 [95% CI 0.74 – 1.03], p = 0.10). Patients with ST-elevation cardiogenic shock without risk of hypoxic brain injury demonstrated reduced 6-month mortality compared to control (HR 0.77 [95% CI 0.61 – 0.97], p = 0.024). |
| Safety findings | All safety events were assessed at 30-days. VA-ECMO and pVAD were associated with increased major bleeding [OR 2.81 (95% CI 1.68 - 4.71) and OR 5.13 (95% CI 1.87 - 14.04)], respectively). Higher rates of stroke (OR 2.25 [95% CI 0.92 – 5.49], p = 0.07), sepsis (OR 2.82 [95% CI 1.49 - 5.33], p = 0.001), urgent dialysis (OR 1.82 [95% CI 1.23 – 2.68], p = 0.003) and limb ischaemia (OR 6.97, [95% CI 1.79 – 27.12] p = 0.005) were noted with pVAD. No significant safety concerns were identified with IABP. | All safety events were assessed at 30-days. MCS was associated with increased major bleeding (OR 2.64 [95% CI 1.91 – 3.65]) and vascular complications (OR 4.43 [95% CI 2.37 – 8.26]). No difference observed for renal replacement therapy (OR 1.29 [95% CI 0.94 – 1.77]), Sepsis (OR 1.28 [95% CI 0.87 – 1.88]) or stroke (OR 1.48 [95% CI 0.72 – 3.04]). |

Supplementary Table 8. Comparison of our pairwise and network meta-analysis with a recent IPD meta-analysis by Thiele et al. (26)

*AMI-CS* Acute myocardial infarction-related cardiogenic shock*, IABP* Intra-aortic balloon pump*,IPD* individual Participant Data, *MCS* Mechanical Circulatory Support, *HR* Hazard Ratio, *OR* Odds Ratio, *CI* Confidence Interval, *pVAD* percutaneous ventricular assist device, *VA-ECMO* Veno-arterial extra-corporeal membrane oxygenation

# Search Strategies

## Medline :

Ovid MEDLINE(R) ALL <1946 to May 13, 2024>

1 Randomized controlled trials as Topic/ 169841

2 Randomized controlled trial/ 612970

3 Random allocation/ 107201

4 Double blind method/ 178474

5 Single blind method/ 33477

6 Clinical trial/ 539899

7 exp Clinical Trials as Topic/ 391932

8 or/1-7 1349146

9 (clinic$ adj trial$1).tw. 510640

10 ((singl$ or doubl$ or treb$ or tripl$) adj (blind$3 or mask$3)).tw. 205035

11 Placebos/ 35949

12 Placebo$.tw. 256216

13 Randomly allocated.tw. 38590

14 (allocated adj2 random).tw. 850

15 or/9-14 823065

16 8 or 15 1736799

17 Case report.tw. 425985

18 Letter/ 1252848

19 Historical article/ 369789

20 Review of reported cases.pt. 0

21 Review, multicase.pt. 0

22 or/17-21 2028575

23 16 not 22 1696638

24 Myocardial Infarction.mp. [mp=title, book title, abstract, original title, name of substance word, subject heading word, floating sub-heading word, keyword heading word, organism supplementary concept word, protocol supplementary concept word, rare disease supplementary concept word, unique identifier, synonyms, population supplementary concept word, anatomy supplementary concept word] 284071

25 Acute MI.mp. [mp=title, book title, abstract, original title, name of substance word, subject heading word, floating sub-heading word, keyword heading word, organism supplementary concept word, protocol supplementary concept word, rare disease supplementary concept word, unique identifier, synonyms, population supplementary concept word, anatomy supplementary concept word] 3820

26 Infarct*.mp. [mp=title, book title, abstract, original title, name of substance word, subject heading word, floating sub-heading word, keyword heading word, organism supplementary concept word, protocol supplementary concept word, rare disease supplementary concept word, unique identifier, synonyms, population supplementary concept word, anatomy supplementary concept word] 397016

27 Heart attack.mp. [mp=title, book title, abstract, original title, name of substance word, subject heading word, floating sub-heading word, keyword heading word, organism supplementary concept word, protocol supplementary concept word, rare disease supplementary concept word, unique identifier, synonyms, population supplementary concept word, anatomy supplementary concept word] 5431

28 AMI-CS.mp. [mp=title, book title, abstract, original title, name of substance word, subject heading word, floating sub-heading word, keyword heading word, organism supplementary concept word, protocol supplementary concept word, rare disease supplementary concept word, unique identifier, synonyms, population supplementary concept word, anatomy supplementary concept word] 152

29 AMICS.mp. [mp=title, book title, abstract, original title, name of substance word, subject heading word, floating sub-heading word, keyword heading word, organism supplementary concept word, protocol supplementary concept word, rare disease supplementary concept word, unique identifier, synonyms, population supplementary concept word, anatomy supplementary concept word] 85

30 STEMI.mp. [mp=title, book title, abstract, original title, name of substance word, subject heading word, floating sub-heading word, keyword heading word, organism supplementary concept word, protocol supplementary concept word, rare disease supplementary concept word, unique identifier, synonyms, population supplementary concept word, anatomy supplementary concept word] 16029

31 ST segment elevation.mp. [mp=title, book title, abstract, original title, name of substance word, subject heading word, floating sub-heading word, keyword heading word, organism supplementary concept word, protocol supplementary concept word, rare disease supplementary concept word, unique identifier, synonyms, population supplementary concept word, anatomy supplementary concept word] 20477

32 (ST adj3 MI).mp. [mp=title, book title, abstract, original title, name of substance word, subject heading word, floating sub-heading word, keyword heading word, organism supplementary concept word, protocol supplementary concept word, rare disease supplementary concept word, unique identifier, synonyms, population supplementary concept word, anatomy supplementary concept word] 1775

33 non ST segment elevation.mp. [mp=title, book title, abstract, original title, name of substance word, subject heading word, floating sub-heading word, keyword heading word, organism supplementary concept word, protocol supplementary concept word, rare disease supplementary concept word, unique identifier, synonyms, population supplementary concept word, anatomy supplementary concept word] 4166

34 NSTEMI.mp. [mp=title, book title, abstract, original title, name of substance word, subject heading word, floating sub-heading word, keyword heading word, organism supplementary concept word, protocol supplementary concept word, rare disease supplementary concept word, unique identifier, synonyms, population supplementary concept word, anatomy supplementary concept word] 3753

35 Acute coronary syndrome.mp. [mp=title, book title, abstract, original title, name of substance word, subject heading word, floating sub-heading word, keyword heading word, organism supplementary concept word, protocol supplementary concept word, rare disease supplementary concept word, unique identifier, synonyms, population supplementary concept word, anatomy supplementary concept word] 37983

36 ACS.mp. [mp=title, book title, abstract, original title, name of substance word, subject heading word, floating sub-heading word, keyword heading word, organism supplementary concept word, protocol supplementary concept word, rare disease supplementary concept word, unique identifier, synonyms, population supplementary concept word, anatomy supplementary concept word] 30996

37 exp Myocardial Infarction/ 197759

38 exp ST Elevation Myocardial Infarction/ 8238

39 exp Coronary Thrombosis/ or exp Acute Coronary Syndrome/ 28944

40 24 or 25 or 26 or 27 or 28 or 29 or 30 or 31 or 32 or 33 or 34 or 35 or 36 or 37 or 38 or 39 448770

41 exp Shock, Cardiogenic/ 11232

42 cardiogenic shock.mp. [mp=title, book title, abstract, original title, name of substance word, subject heading word, floating sub-heading word, keyword heading word, organism supplementary concept word, protocol supplementary concept word, rare disease supplementary concept word, unique identifier, synonyms, population supplementary concept word, anatomy supplementary concept word] 16271

43 ((cardiogenic or cardiac or circulatory or heart or cardiovascular or myocard*) adj2 (shock* or failure)).mp. [mp=title, book title, abstract, original title, name of substance word, subject heading word, floating sub-heading word, keyword heading word, organism supplementary concept word, protocol supplementary concept word, rare disease supplementary concept word, unique identifier, synonyms, population supplementary concept word, anatomy supplementary concept word] 298988

44 ((Cardiogenic or Circulat*) adj4 (shock or failure)).mp. [mp=title, book title, abstract, original title, name of substance word, subject heading word, floating sub-heading word, keyword heading word, organism supplementary concept word, protocol supplementary concept word, rare disease supplementary concept word, unique identifier, synonyms, population supplementary concept word, anatomy supplementary concept word] 26126

45 Shock.mp. [mp=title, book title, abstract, original title, name of substance word, subject heading word, floating sub-heading word, keyword heading word, organism supplementary concept word, protocol supplementary concept word, rare disease supplementary concept word, unique identifier, synonyms, population supplementary concept word, anatomy supplementary concept word] 268348

46 41 or 42 or 43 or 44 or 45 540649

47 (Mechanical adj2 support).mp. [mp=title, book title, abstract, original title, name of substance word, subject heading word, floating sub-heading word, keyword heading word, organism supplementary concept word, protocol supplementary concept word, rare disease supplementary concept word, unique identifier, synonyms, population supplementary concept word, anatomy supplementary concept word] 12633

48 VA-ECMO.mp. [mp=title, book title, abstract, original title, name of substance word, subject heading word, floating sub-heading word, keyword heading word, organism supplementary concept word, protocol supplementary concept word, rare disease supplementary concept word, unique identifier, synonyms, population supplementary concept word, anatomy supplementary concept word] 1998

49 ECMO.mp. [mp=title, book title, abstract, original title, name of substance word, subject heading word, floating sub-heading word, keyword heading word, organism supplementary concept word, protocol supplementary concept word, rare disease supplementary concept word, unique identifier, synonyms, population supplementary concept word, anatomy supplementary concept word] 14083

50 ECLS.mp. [mp=title, book title, abstract, original title, name of substance word, subject heading word, floating sub-heading word, keyword heading word, organism supplementary concept word, protocol supplementary concept word, rare disease supplementary concept word, unique identifier, synonyms, population supplementary concept word, anatomy supplementary concept word] 1942

51 ((Extracorporeal or extra corporeal or extrapulmonary or extra pulmonary) adj3 oxygen*).mp. [mp=title, book title, abstract, original title, name of substance word, subject heading word, floating sub-heading word, keyword heading word, organism supplementary concept word, protocol supplementary concept word, rare disease supplementary concept word, unique identifier, synonyms, population supplementary concept word, anatomy supplementary concept word] 24326

52 Extracorporeal life support.mp. [mp=title, book title, abstract, original title, name of substance word, subject heading word, floating sub-heading word, keyword heading word, organism supplementary concept word, protocol supplementary concept word, rare disease supplementary concept word, unique identifier, synonyms, population supplementary concept word, anatomy supplementary concept word] 3264

53 Impella.mp. [mp=title, book title, abstract, original title, name of substance word, subject heading word, floating sub-heading word, keyword heading word, organism supplementary concept word, protocol supplementary concept word, rare disease supplementary concept word, unique identifier, synonyms, population supplementary concept word, anatomy supplementary concept word] 1787

54 IABP.mp. [mp=title, book title, abstract, original title, name of substance word, subject heading word, floating sub-heading word, keyword heading word, organism supplementary concept word, protocol supplementary concept word, rare disease supplementary concept word, unique identifier, synonyms, population supplementary concept word, anatomy supplementary concept word] 2731

55 Intra-aortic balloon pump*.mp. [mp=title, book title, abstract, original title, name of substance word, subject heading word, floating sub-heading word, keyword heading word, organism supplementary concept word, protocol supplementary concept word, rare disease supplementary concept word, unique identifier, synonyms, population supplementary concept word, anatomy supplementary concept word] 6761

56 (Intra-aortic and (pump or balloon)).mp. [mp=title, book title, abstract, original title, name of substance word, subject heading word, floating sub-heading word, keyword heading word, organism supplementary concept word, protocol supplementary concept word, rare disease supplementary concept word, unique identifier, synonyms, population supplementary concept word, anatomy supplementary concept word] 7428

57 (Balloon adj2 pump*).mp. [mp=title, book title, abstract, original title, name of substance word, subject heading word, floating sub-heading word, keyword heading word, organism supplementary concept word, protocol supplementary concept word, rare disease supplementary concept word, unique identifier, synonyms, population supplementary concept word, anatomy supplementary concept word] 7717

58 (heart adj2 pump).mp. [mp=title, book title, abstract, original title, name of substance word, subject heading word, floating sub-heading word, keyword heading word, organism supplementary concept word, protocol supplementary concept word, rare disease supplementary concept word, unique identifier, synonyms, population supplementary concept word, anatomy supplementary concept word] 826

59 LVAD.mp. [mp=title, book title, abstract, original title, name of substance word, subject heading word, floating sub-heading word, keyword heading word, organism supplementary concept word, protocol supplementary concept word, rare disease supplementary concept word, unique identifier, synonyms, population supplementary concept word, anatomy supplementary concept word] 6767

60 pVAD.mp. [mp=title, book title, abstract, original title, name of substance word, subject heading word, floating sub-heading word, keyword heading word, organism supplementary concept word, protocol supplementary concept word, rare disease supplementary concept word, unique identifier, synonyms, population supplementary concept word, anatomy supplementary concept word] 226

61 VAD.mp. [mp=title, book title, abstract, original title, name of substance word, subject heading word, floating sub-heading word, keyword heading word, organism supplementary concept word, protocol supplementary concept word, rare disease supplementary concept word, unique identifier, synonyms, population supplementary concept word, anatomy supplementary concept word] 10641

62 ((Ventric* or percutaneous or heart or vascular or cardiac or LV) adj3 assist adj2 (device* or pump*)).mp. [mp=title, book title, abstract, original title, name of substance word, subject heading word, floating sub-heading word, keyword heading word, organism supplementary concept word, protocol supplementary concept word, rare disease supplementary concept word, unique identifier, synonyms, population supplementary concept word, anatomy supplementary concept word] 23112

63 (Artificial adj2 ventric*).mp. [mp=title, book title, abstract, original title, name of substance word, subject heading word, floating sub-heading word, keyword heading word, organism supplementary concept word, protocol supplementary concept word, rare disease supplementary concept word, unique identifier, synonyms, population supplementary concept word, anatomy supplementary concept word] 219

64 ((mechanical or ventricular) and assist device).mp. [mp=title, book title, abstract, original title, name of substance word, subject heading word, floating sub-heading word, keyword heading word, organism supplementary concept word, protocol supplementary concept word, rare disease supplementary concept word, unique identifier, synonyms, population supplementary concept word, anatomy supplementary concept word] 13985

65 HeartMate.mp. [mp=title, book title, abstract, original title, name of substance word, subject heading word, floating sub-heading word, keyword heading word, organism supplementary concept word, protocol supplementary concept word, rare disease supplementary concept word, unique identifier, synonyms, population supplementary concept word, anatomy supplementary concept word] 1888

66 CentriMag.mp. [mp=title, book title, abstract, original title, name of substance word, subject heading word, floating sub-heading word, keyword heading word, organism supplementary concept word, protocol supplementary concept word, rare disease supplementary concept word, unique identifier, synonyms, population supplementary concept word, anatomy supplementary concept word] 219

67 Counterpulsation.mp. [mp=title, book title, abstract, original title, name of substance word, subject heading word, floating sub-heading word, keyword heading word, organism supplementary concept word, protocol supplementary concept word, rare disease supplementary concept word, unique identifier, synonyms, population supplementary concept word, anatomy supplementary concept word] 2495

68 TandemHeart.mp. [mp=title, book title, abstract, original title, name of substance word, subject heading word, floating sub-heading word, keyword heading word, organism supplementary concept word, protocol supplementary concept word, rare disease supplementary concept word, unique identifier, synonyms, population supplementary concept word, anatomy supplementary concept word] 235

69 Tandem heart.mp. [mp=title, book title, abstract, original title, name of substance word, subject heading word, floating sub-heading word, keyword heading word, organism supplementary concept word, protocol supplementary concept word, rare disease supplementary concept word, unique identifier, synonyms, population supplementary concept word, anatomy supplementary concept word] 41

70 Heart-Assist Device*.mp. [mp=title, book title, abstract, original title, name of substance word, subject heading word, floating sub-heading word, keyword heading word, organism supplementary concept word, protocol supplementary concept word, rare disease supplementary concept word, unique identifier, synonyms, population supplementary concept word, anatomy supplementary concept word] 18627

71 ((Microaxial or Axial) adj3 pump*).mp. [mp=title, book title, abstract, original title, name of substance word, subject heading word, floating sub-heading word, keyword heading word, organism supplementary concept word, protocol supplementary concept word, rare disease supplementary concept word, unique identifier, synonyms, population supplementary concept word, anatomy supplementary concept word] 765

72 (Centrifugal adj3 pump*).mp. [mp=title, book title, abstract, original title, name of substance word, subject heading word, floating sub-heading word, keyword heading word, organism supplementary concept word, protocol supplementary concept word, rare disease supplementary concept word, unique identifier, synonyms, population supplementary concept word, anatomy supplementary concept word] 1970

73 Assisted circulation.mp. [mp=title, book title, abstract, original title, name of substance word, subject heading word, floating sub-heading word, keyword heading word, organism supplementary concept word, protocol supplementary concept word, rare disease supplementary concept word, unique identifier, synonyms, population supplementary concept word, anatomy supplementary concept word] 3767

74 ((hemodynamic* or haemodynamic* or circulat* or mechanic*) adj3 (device* or support*)).mp. [mp=title, book title, abstract, original title, name of substance word, subject heading word, floating sub-heading word, keyword heading word, organism supplementary concept word, protocol supplementary concept word, rare disease supplementary concept word, unique identifier, synonyms, population supplementary concept word, anatomy supplementary concept word] 26609

75 exp Assisted Circulation/ or exp Heart-Assist Devices/ or exp Extracorporeal Membrane Oxygenation/ 39449

76 exp Intra-Aortic Balloon Pumping/ 4412

77 47 or 48 or 49 or 50 or 51 or 52 or 53 or 54 or 55 or 56 or 57 or 58 or 59 or 60 or 61 or 62 or 63 or 64 or 65 or 66 or 67 or 68 or 69 or 70 or 71 or 72 or 73 or 74 or 75 or 76 83395

78 23 and 40 and 46 and 77 480

79 limit 78 to english language 449

80 from 79 keep 1-449 449

## CENTRAL (Cochrane library) :

Search Name: AMICS_CENTRAL

Date Run: 14/05/2024 16:17:19

Comment:

ID Search Hits

#1 "Myocardial Infarction" 37705

#2 "Acute MI" 724

#3 Infarct* 50782

#4 “AMI CS” 10

#5 AMICS 27

#6 STEMI 4250

#7 “ST segment elevation” 6397

#8 “ST" NEAR/3 "MI” 578

#9 “non ST segment elevation” 1645

#10 NSTEMI 820

#11 “Acute Coronary Syndrome” 8029

#12 ACS 6063

#13 “Heart attack” 1501

#14 MeSH descriptor: [Myocardial Infarction] explode all trees 15664

#15 MeSH descriptor: [ST Elevation Myocardial Infarction] explode all trees 1128

#16 MeSH descriptor: [Angina, Unstable] explode all trees 1457

#17 MeSH descriptor: [Non-ST Elevated Myocardial Infarction] explode all trees 191

#18 MeSH descriptor: [Coronary Thrombosis] explode all trees 668

#19 MeSH descriptor: [Acute Coronary Syndrome] explode all trees 3118

#20 #1 OR #2 OR #3 OR #4 OR #5 OR #6 OR #7 OR #8 OR #9 OR #10 OR #11 OR #12 OR #13 OR #14 OR #15 OR #16 OR #17 OR #18 OR #19 58159

#21 “Cardiogenic Shock” 1659

#22 Shock 15015

#23 (cardiogenic OR circulation OR circulatory) NEAR/4 (shock OR failure) 2632

#24 (cardiogenic OR cardiac OR circulatory OR heart OR cardiovascular OR myocardial OR myocardium) NEAR/2 (shock OR failure) 41739

#25 MeSH descriptor: [Shock, Cardiogenic] explode all trees 482

#26 #21 OR #22 OR #23 OR #24 OR #25 54132

#27 MeSH descriptor: [Assisted Circulation] explode all trees 664

#28 MeSH descriptor: [Heart-Assist Devices] explode all trees 380

#29 MeSH descriptor: [Extracorporeal Membrane Oxygenation] explode all trees 356

#30 MeSH descriptor: [Intra-Aortic Balloon Pumping] explode all trees 228

#31 “Mechanical" NEAR/2 "support” 1143

#32 “VA ECMO” 113

#33 ECMO 1067

#34 ECLS 74

#35 (Extracorporeal OR "extra corporeal" OR extrapulmonary OR "extra pulmonary") NEAR/3 (oxygen OR oxygenator OR oxygenation) 1266

#36 “Extracorporeal Life Support” 100

#37 Impella 157

#38 IABP 400

#39 “Intra aortic balloon pump” 366

#40 "Intra aortic balloon pumping" 246

#41 (Intraaortic) AND (pump OR balloon) 684

#42 balloon NEAR/2 (pump OR pumping) 684

#43 Heart NEAR/2 pump 189

#44 LVAD 490

#45 pVAD 19

#46 VAD 653

#47 (Ventricular OR ventricle OR percutaneous OR heart OR vascular OR cardiac OR LV) NEAR/3 assist NEAR/2 (device OR devices OR pump OR pumping) 983

#48 Artificial NEAR/2 ventric* 71

#49 (mechanical OR ventricular OR ventricular) AND (assist device) 1046

#50 HeartMate 148

#51 CentriMag 2

#52 Counterpulsation 392

#53 TandemHeart 14

#54 “Tandem Heart” 16

#55 (Microaxial OR Axial) NEAR/3 (pump OR pumping) 38

#56 “Heart Assist Device” 71

#57 Centrifugal NEAR/3 pump* 121

#58 “Assisted circulation” 272

#59 (hemodynamic OR haemodynamic OR haemdynamically OR hemodynamically OR circulatory OR circulation OR mechanic OR mechanical) NEAR/3 (device OR support OR supporting OR supportive) 2135

#60 #27 OR #28 OR #29 OR #30 OR #31 OR #32 OR #33 OR #34 OR #35 OR #36 OR #37 OR #38 OR #39 OR #40 OR #41 OR #42 OR #43 OR #44 OR #45 OR #46 OR #47 OR #48 OR #49 OR #50 OR #51 OR #52 OR #53 OR #54 OR #55 OR #56 OR #57 OR #58 OR #59 6472

#61 #20 AND #26 AND #60 in Trials 530

## Embase :

Embase Classic+Embase <1947 to 2024 May 13>

1 Clinical trial/ 1103373

2 Randomized controlled trial/ 824438

3 Randomization/ 99572

4 Single blind procedure/ 54778

5 Double blind procedure/ 221580

6 Crossover procedure/ 78365

7 Placebo/ 423465

8 Randomi?ed controlled trial$.tw. 346011

9 Rct.tw. 57771

10 Random allocation.tw. 2749

11 Randomly allocated.tw. 47752

12 Allocated randomly.tw. 3067

13 (allocated adj2 random).tw. 1049

14 Single blind$.tw. 33274

15 Double blind$.tw. 257468

16 ((treble or triple) adj blind$).tw. 2139

17 Placebo$.tw. 384576

18 Prospective study/ 920757

19 or/1-18 2902177

20 Case study/ 110329

21 Case report.tw. 596851

22 Abstract report/ or letter/ 1329439

23 or/20-22 2020510

24 19 not 23 2834314

25 Myocardial Infarction.mp. [mp=title, abstract, heading word, drug trade name, original title, device manufacturer, drug manufacturer, device trade name, keyword heading word, floating subheading word, candidate term word] 367632

26 Acute MI.mp. [mp=title, abstract, heading word, drug trade name, original title, device manufacturer, drug manufacturer, device trade name, keyword heading word, floating subheading word, candidate term word] 7286

27 Infarct*.mp. [mp=title, abstract, heading word, drug trade name, original title, device manufacturer, drug manufacturer, device trade name, keyword heading word, floating subheading word, candidate term word] 712667

28 Heart attack.mp. [mp=title, abstract, heading word, drug trade name, original title, device manufacturer, drug manufacturer, device trade name, keyword heading word, floating subheading word, candidate term word] 8267

29 AMI-CS.mp. [mp=title, abstract, heading word, drug trade name, original title, device manufacturer, drug manufacturer, device trade name, keyword heading word, floating subheading word, candidate term word] 305

30 AMICS.mp. [mp=title, abstract, heading word, drug trade name, original title, device manufacturer, drug manufacturer, device trade name, keyword heading word, floating subheading word, candidate term word] 174

31 STEMI.mp. [mp=title, abstract, heading word, drug trade name, original title, device manufacturer, drug manufacturer, device trade name, keyword heading word, floating subheading word, candidate term word] 38489

32 ST segment elevation.mp. [mp=title, abstract, heading word, drug trade name, original title, device manufacturer, drug manufacturer, device trade name, keyword heading word, floating subheading word, candidate term word] 101463

33 (ST adj3 MI).mp. [mp=title, abstract, heading word, drug trade name, original title, device manufacturer, drug manufacturer, device trade name, keyword heading word, floating subheading word, candidate term word] 4015

34 non ST segment elevation.mp. [mp=title, abstract, heading word, drug trade name, original title, device manufacturer, drug manufacturer, device trade name, keyword heading word, floating subheading word, candidate term word] 27165

35 NSTEMI.mp. [mp=title, abstract, heading word, drug trade name, original title, device manufacturer, drug manufacturer, device trade name, keyword heading word, floating subheading word, candidate term word] 10773

36 Acute coronary syndrome.mp. [mp=title, abstract, heading word, drug trade name, original title, device manufacturer, drug manufacturer, device trade name, keyword heading word, floating subheading word, candidate term word] 89901

37 ACS.mp. [mp=title, abstract, heading word, drug trade name, original title, device manufacturer, drug manufacturer, device trade name, keyword heading word, floating subheading word, candidate term word] 58563

38 exp heart infarction/ 491088

39 ST segment elevation myocardial infarction/ or exp unstable angina pectoris/ 79673

40 exp coronary artery thrombosis/ or exp acute coronary syndrome/ or exp acute heart infarction/ 172547

41 25 or 26 or 27 or 28 or 29 or 30 or 31 or 32 or 33 or 34 or 35 or 36 or 37 or 38 or 39 or 40 807360

42 exp shock/ or exp cardiogenic shock/ 190848

43 cardiogenic shock.mp. [mp=title, abstract, heading word, drug trade name, original title, device manufacturer, drug manufacturer, device trade name, keyword heading word, floating subheading word, candidate term word] 45725

44 ((cardiogenic or cardiac or circulatory or heart or cardiovascular or myocard*) adj2 (shock* or failure)).mp. [mp=title, abstract, heading word, drug trade name, original title, device manufacturer, drug manufacturer, device trade name, keyword heading word, floating subheading word, candidate term word] 604495

45 ((Cardiogenic or Circulat*) adj4 (shock or failure)).mp. [mp=title, abstract, heading word, drug trade name, original title, device manufacturer, drug manufacturer, device trade name, keyword heading word, floating subheading word, candidate term word] 56195

46 Shock.mp. [mp=title, abstract, heading word, drug trade name, original title, device manufacturer, drug manufacturer, device trade name, keyword heading word, floating subheading word, candidate term word] 442541

47 42 or 43 or 44 or 45 or 46 989146

48 (Mechanical adj2 support).mp. [mp=title, abstract, heading word, drug trade name, original title, device manufacturer, drug manufacturer, device trade name, keyword heading word, floating subheading word, candidate term word] 21415

49 VA-ECMO.mp. [mp=title, abstract, heading word, drug trade name, original title, device manufacturer, drug manufacturer, device trade name, keyword heading word, floating subheading word, candidate term word] 4980

50 ECMO.mp. [mp=title, abstract, heading word, drug trade name, original title, device manufacturer, drug manufacturer, device trade name, keyword heading word, floating subheading word, candidate term word] 31762

51 ECLS.mp. [mp=title, abstract, heading word, drug trade name, original title, device manufacturer, drug manufacturer, device trade name, keyword heading word, floating subheading word, candidate term word] 3370

52 ((Extracorporeal or extra corporeal or extrapulmonary or extra pulmonary) adj3 oxygen*).mp. [mp=title, abstract, heading word, drug trade name, original title, device manufacturer, drug manufacturer, device trade name, keyword heading word, floating subheading word, candidate term word] 49074

53 Extracorporeal life support.mp. [mp=title, abstract, heading word, drug trade name, original title, device manufacturer, drug manufacturer, device trade name, keyword heading word, floating subheading word, candidate term word] 5136

54 Impella.mp. [mp=title, abstract, heading word, drug trade name, original title, device manufacturer, drug manufacturer, device trade name, keyword heading word, floating subheading word, candidate term word] 5382

55 IABP.mp. [mp=title, abstract, heading word, drug trade name, original title, device manufacturer, drug manufacturer, device trade name, keyword heading word, floating subheading word, candidate term word] 6217

56 Intra-aortic balloon pump*.mp. [mp=title, abstract, heading word, drug trade name, original title, device manufacturer, drug manufacturer, device trade name, keyword heading word, floating subheading word, candidate term word] 7200

57 (Intra-aortic and (pump or balloon)).mp. [mp=title, abstract, heading word, drug trade name, original title, device manufacturer, drug manufacturer, device trade name, keyword heading word, floating subheading word, candidate term word] 8850

58 (Balloon adj2 pump*).mp. [mp=title, abstract, heading word, drug trade name, original title, device manufacturer, drug manufacturer, device trade name, keyword heading word, floating subheading word, candidate term word] 14800

59 (heart adj2 pump).mp. [mp=title, abstract, heading word, drug trade name, original title, device manufacturer, drug manufacturer, device trade name, keyword heading word, floating subheading word, candidate term word] 1308

60 LVAD.mp. [mp=title, abstract, heading word, drug trade name, original title, device manufacturer, drug manufacturer, device trade name, keyword heading word, floating subheading word, candidate term word] 17229

61 pVAD.mp. [mp=title, abstract, heading word, drug trade name, original title, device manufacturer, drug manufacturer, device trade name, keyword heading word, floating subheading word, candidate term word] 589

62 VAD.mp. [mp=title, abstract, heading word, drug trade name, original title, device manufacturer, drug manufacturer, device trade name, keyword heading word, floating subheading word, candidate term word] 17759

63 ((Ventric* or percutaneous or heart or vascular or cardiac or LV) adj3 assist adj2 (device* or pump*)).mp. [mp=title, abstract, heading word, drug trade name, original title, device manufacturer, drug manufacturer, device trade name, keyword heading word, floating subheading word, candidate term word] 43927

64 (Artificial adj2 ventric*).mp. [mp=title, abstract, heading word, drug trade name, original title, device manufacturer, drug manufacturer, device trade name, keyword heading word, floating subheading word, candidate term word] 334

65 ((mechanical or ventricular) and assist device).mp. [mp=title, abstract, heading word, drug trade name, original title, device manufacturer, drug manufacturer, device trade name, keyword heading word, floating subheading word, candidate term word] 40767

66 HeartMate.mp. [mp=title, abstract, heading word, drug trade name, original title, device manufacturer, drug manufacturer, device trade name, keyword heading word, floating subheading word, candidate term word] 7231

67 CentriMag.mp. [mp=title, abstract, heading word, drug trade name, original title, device manufacturer, drug manufacturer, device trade name, keyword heading word, floating subheading word, candidate term word] 1197

68 Counterpulsation.mp. [mp=title, abstract, heading word, drug trade name, original title, device manufacturer, drug manufacturer, device trade name, keyword heading word, floating subheading word, candidate term word] 4780

69 TandemHeart.mp. [mp=title, abstract, heading word, drug trade name, original title, device manufacturer, drug manufacturer, device trade name, keyword heading word, floating subheading word, candidate term word] 846

70 Tandem heart.mp. [mp=title, abstract, heading word, drug trade name, original title, device manufacturer, drug manufacturer, device trade name, keyword heading word, floating subheading word, candidate term word] 219

71 Heart-Assist Device*.mp. [mp=title, abstract, heading word, drug trade name, original title, device manufacturer, drug manufacturer, device trade name, keyword heading word, floating subheading word, candidate term word] 8926

72 ((Microaxial or Axial) adj3 pump*).mp. [mp=title, abstract, heading word, drug trade name, original title, device manufacturer, drug manufacturer, device trade name, keyword heading word, floating subheading word, candidate term word] 1332

73 (Centrifugal adj3 pump*).mp. [mp=title, abstract, heading word, drug trade name, original title, device manufacturer, drug manufacturer, device trade name, keyword heading word, floating subheading word, candidate term word] 4817

74 Assisted circulation.mp. [mp=title, abstract, heading word, drug trade name, original title, device manufacturer, drug manufacturer, device trade name, keyword heading word, floating subheading word, candidate term word] 19016

75 ((hemodynamic* or haemodynamic* or circulat* or mechanic*) adj3 (device* or support*)).mp. [mp=title, abstract, heading word, drug trade name, original title, device manufacturer, drug manufacturer, device trade name, keyword heading word, floating subheading word, candidate term word] 42917

76 exp assisted circulation/ or exp ventricular assist device/ or exp veno-arterial ECMO/ or exp microaxial pump/ or exp microaxial pump/ 48431

77 exp intraaortic balloon pump/ 9627

78 48 or 49 or 50 or 51 or 52 or 53 or 54 or 55 or 56 or 57 or 58 or 59 or 60 or 61 or 62 or 63 or 64 or 65 or 66 or 67 or 68 or 69 or 70 or 71 or 72 or 73 or 74 or 75 or 76 or 77 154601

79 24 and 41 and 47 and 78 1343

80 limit 79 to english language 1307

## Scopus :

( ( TITLE-ABS-KEY ( ( h?modynamic* OR circulat* OR mechanic* ) W/3 ( device* OR support* ) ) ) OR ( TITLE-ABS-KEY ( "assisted circulation" ) ) OR ( TITLE-ABS-KEY ( centrifugal W/3 pump* ) ) OR ( TITLE-ABS-KEY ( ( microaxial OR axial ) W/3 pump* ) ) OR ( TITLE-ABS-KEY ( "heart assist device*" ) ) OR ( TITLE-ABS-KEY ( "tandem heart" ) ) OR ( TITLE-ABS-KEY ( tandemheart ) ) OR ( TITLE-ABS-KEY ( counterpulsation ) ) OR ( TITLE-ABS-KEY ( centrimag ) ) OR ( TITLE-ABS-KEY ( heartmate ) ) OR ( TITLE-ABS-KEY ( ( mechanical OR ventricular ) AND "assist device" ) ) OR ( TITLE-ABS-KEY ( artificial W/2 ventric* ) ) OR ( TITLE-ABS-KEY ( ( ventric* OR percutaneous OR heart OR vascular OR cardiac OR lv ) W/3 assist W/2 ( device* OR pump* ) ) ) OR ( TITLE-ABS-KEY ( vad ) ) OR ( TITLE-ABS-KEY ( pvad ) ) OR ( TITLE-ABS-KEY ( lvad ) ) OR ( TITLE-ABS-KEY ( heart W/2 pump ) ) OR ( TITLE-ABS-KEY ( balloon W/2 pump* ) ) OR ( TITLE-ABS-KEY ( "intra aortic" AND ( pump OR balloon ) ) ) OR ( TITLE-ABS-KEY ( "intra aortic balloon pump*" ) ) OR ( TITLE-ABS-KEY ( iabp ) ) OR ( TITLE-ABS-KEY ( impella ) ) OR ( TITLE-ABS-KEY ( "extracorporeal life support" ) ) OR ( TITLE-ABS-KEY ( ( extracorporeal OR "extra corporeal" OR extrapulmonary OR "extra pulmonary" ) W/3 oxygen* ) ) OR ( TITLE-ABS-KEY ( ecls ) ) OR ( TITLE-ABS-KEY ( ecmo ) ) OR ( TITLE-ABS-KEY ( "va ecmo" ) ) OR ( TITLE-ABS-KEY ( mechanical W/2 support ) ) ) AND ( ( TITLE-ABS-KEY ( allocated W/2 random ) ) OR ( TITLE-ABS-KEY ( "randomly allocated" ) ) OR ( TITLE-ABS-KEY ( placebo* ) ) OR ( TITLE-ABS-KEY ( ( singl* OR doubl* OR treb* OR tripl* ) W/2 ( blind* OR mask* ) ) ) OR ( TITLE-ABS-KEY ( clinic* W/3 trial* ) ) OR ( TITLE-ABS-KEY ( "single blind method" ) ) OR ( TITLE-ABS-KEY ( "double blind method" ) ) OR ( TITLE-ABS-KEY ( "random allocation" ) ) OR ( TITLE-ABS-KEY ( "clinical trial*" ) ) OR ( TITLE-ABS-KEY ( "randomized controlled trial*" ) ) ) AND ( ( TITLE-ABS-KEY ( shock ) ) OR ( TITLE-ABS-KEY ( ( cardiogenic OR circulat* ) W/4 ( shock OR failure ) ) ) OR ( TITLE-ABS-KEY ( ( cardiogenic OR cardiac OR circulatory OR heart OR cardiovascular OR myocard* ) W/2 ( shock* OR failure ) ) ) OR ( TITLE-ABS-KEY ( "cardiogenic shock" ) ) ) AND ( ( TITLE-ABS-KEY ( acs ) ) OR ( TITLE-ABS-KEY ( "acute coronary syndrome" ) ) OR ( TITLE-ABS-KEY ( nstemi ) ) OR ( TITLE-ABS-KEY ( "non st segment elevation" ) ) OR ( TITLE-ABS-KEY ( st W/3 mi ) ) OR ( TITLE-ABS-KEY ( "st segment elevation" ) ) OR ( TITLE-ABS-KEY ( stemi ) ) OR ( TITLE-ABS-KEY ( amics ) ) OR ( TITLE-ABS-KEY ( "ami-cs" ) ) OR ( TITLE-ABS-KEY ( "heart attack" ) ) OR ( TITLE-ABS-KEY ( infarct* ) ) OR ( "myocardial infarction" ) ) AND ( LIMIT-TO ( LANGUAGE , "english" ) )

## Web of Science :

# Web of Science Search Strategy (v0.1)

# Database: Web of Science Core Collection

# Entitlements:

- WOS.IC: 1993 to 2024

- WOS.CCR: 1985 to 2024

- WOS.SCI: 1900 to 2024

- WOS.AHCI: 1975 to 2024

- WOS.BHCI: 2005 to 2024

- WOS.BSCI: 2005 to 2024

- WOS.ESCI: 2005 to 2024

- WOS.ISTP: 1990 to 2024

- WOS.SSCI: 1900 to 2024

- WOS.ISSHP: 1990 to 2024

# Searches:

1: TS=(allocated NEAR/2 random) Results: 847

2: TS=("Randomly allocated") Results: 39209

3: TS=(Placebo*) Results: 316781

4: TS=((singl$ or doubl$ or treb$ or tripl$) NEAR (blind* or mask*)) Results: 402380

5: TS=(clinic$ NEAR trial$) Results: 9231

6: TS=("Single blind method") Results: 72

7: TS=("Double blind method") Results: 507

8: TS=("Random allocation") Results: 2672

9: TS=("Clinical Trial*") Results: 591360

10: TS=("Randomi?ed controlled trial*") Results: 376930

11: #1 OR #2 OR #3 OR #4 OR #5 OR #6 OR #7 OR #8 OR #9 OR #10 Results: 1320670

12: TS=(ACS) Results: 45673

13: TS=(“Acute Coronary Syndrome”) Results: 41174

14: TS=(NSTEMI) Results: 4273

15: TS=(“non ST segment elevation”) Results: 4696

16: TS=(ST NEAR/3 MI) Results: 2115

17: TS=(“ST segment elevation”) Results: 27710

18: TS=(STEMI) Results: 19480

19: TS=(AMICS) Results: 123

20: TS=(“AMI-CS”) Results: 139

21: TS=(“Heart attack”) Results: 6285

22: TS=(Infarct*) Results: 479533

23: TS=(“Acute MI”) Results: 3676

24: TS=(“Myocardial Infarction”) Results: 350198

25: #12 OR #13 OR #14 OR #15 OR #16 OR #17 OR #18 OR #19 OR #20 OR #21 OR #22 OR #23 OR #24 Results: 540757

26: TS=(Shock) Results: 515340

27: TS=((Cardiogenic OR circulat*) NEAR/4 (shock OR failure)) Results: 26201

28: TS=((cardiogenic OR cardiac OR circulatory OR heart OR cardiovascular OR myocard*) NEAR/2 (shock* OR failure)) Results: 371573

29: TS=(“Cardiogenic Shock”) Results: 18873

30: #26 OR #27 OR #28 OR #29 Results: 858712

31: TS=(((Ventric* OR percutaneous OR heart OR vascular OR cardiac OR LV) NEAR/3 (assist) NEAR/2 (device* OR pump*))) Results: 24422

32: TS=((h?modynamic* OR circulat* OR mechanic*) NEAR/3 (device* or support*)) Results: 47756

33: TS=("Assisted circulation") Results: 508

34: TS=(Centrifugal NEAR/3 pump*) Results: 8125

35: TS=((Microaxial OR Axial) NEAR/3 pump*) Results: 2879

36: TS=("Heart Assist Device*") Results: 1001

37: TS=("Tandem Heart") Results: 66

38: TS=(TandemHeart) Results: 235

39: TS=(Counterpulsation) Results: 3617

40: TS=(CentriMag) Results: 298

41: TS=(HeartMate) Results: 2377

42: TS=((mechanical OR ventricular) AND ("assist device”)) Results: 20216

43: TS=(Artificial NEAR/2 ventric*) Results: 314

44: TS=(VAD) Results: 12160

45: TS=(pVAD) Results: 249

46: TS=(LVAD) Results: 8879

47: TS=(Heart NEAR/2 pump) Results: 2080

48: TS=(balloon NEAR/2 pump*) Results: 5836

49: TS=("Intra aortic" AND (pump OR balloon)) Results: 4929

50: TS=(“Intra aortic balloon pump*”) Results: 3843

51: TS=(IABP) Results: 2319

52: TS=(Impella) Results: 2424

53: TS=("Extracorporeal Life Support") Results: 3938

54: TS=((Extracorporeal OR "extra corporeal" OR extrapulmonary OR "extra pulmonary") NEAR/3 oxygen*) Results: 24232

55: TS=(ECLS) Results: 2595

56: TS=(ECMO) Results: 16029

57: TS=("VA ECMO") Results: 2107

58: TS=(Mechanical NEAR/2 Support) Results: 19750

59: #31 OR #32 OR #33 OR #34 OR #35 OR #36 OR #37 OR #38 OR #39 OR #40 OR #41 OR #42 OR #43 OR #44 OR #45 OR #46 OR #47 OR #48 OR #49 OR #50 OR #51 OR #52 OR #53 OR #54 OR #55 OR #56 OR #57 OR #58 Results: 120521

60: #11 AND #25 AND #30 AND #59 and English (Languages) Results: 495


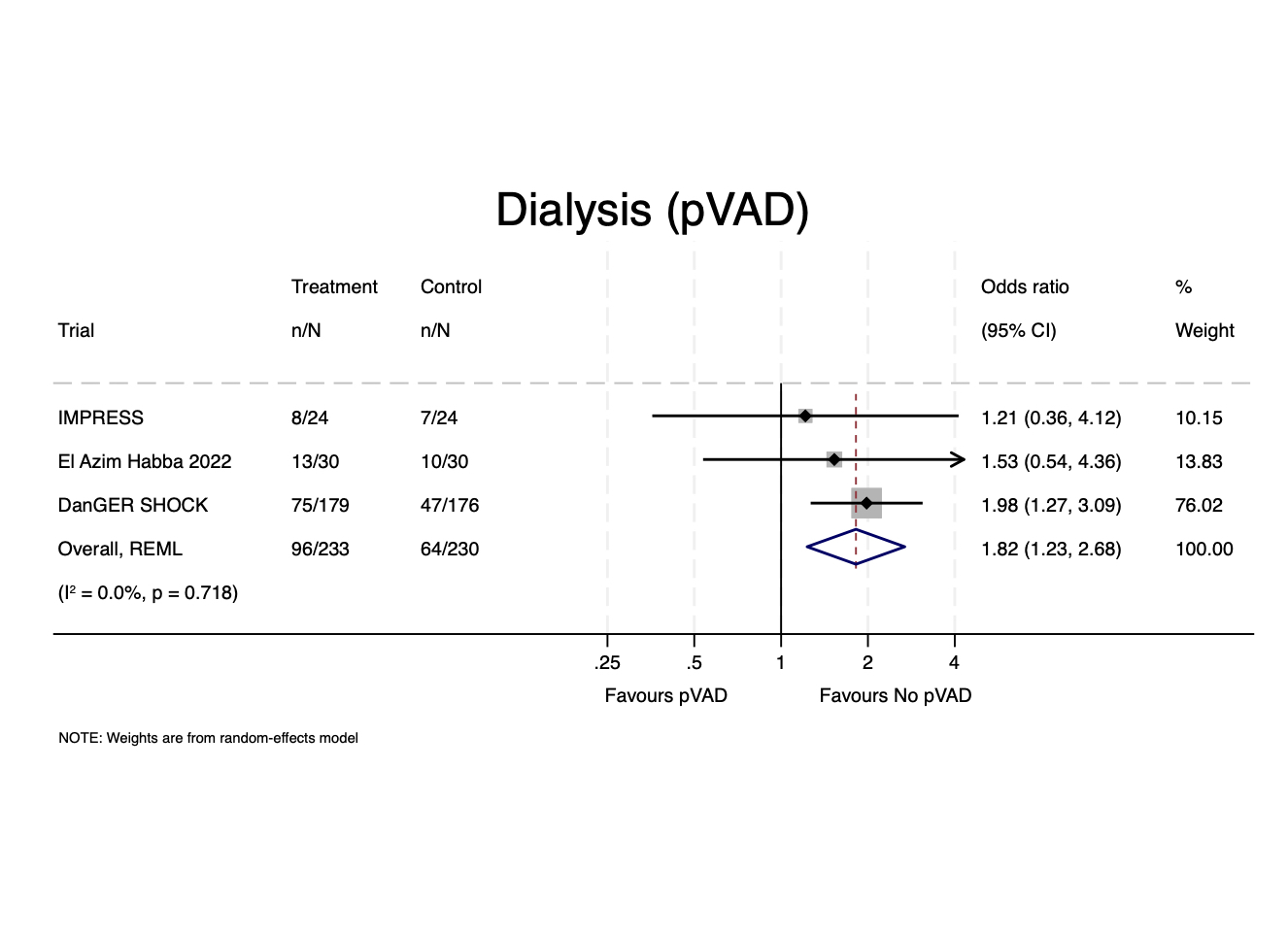


# Supplementary Figure 1. Urgent dialysis (pVAD)

*pVAD* Percutaneous ventricular assist device, *CI* Confidence interval


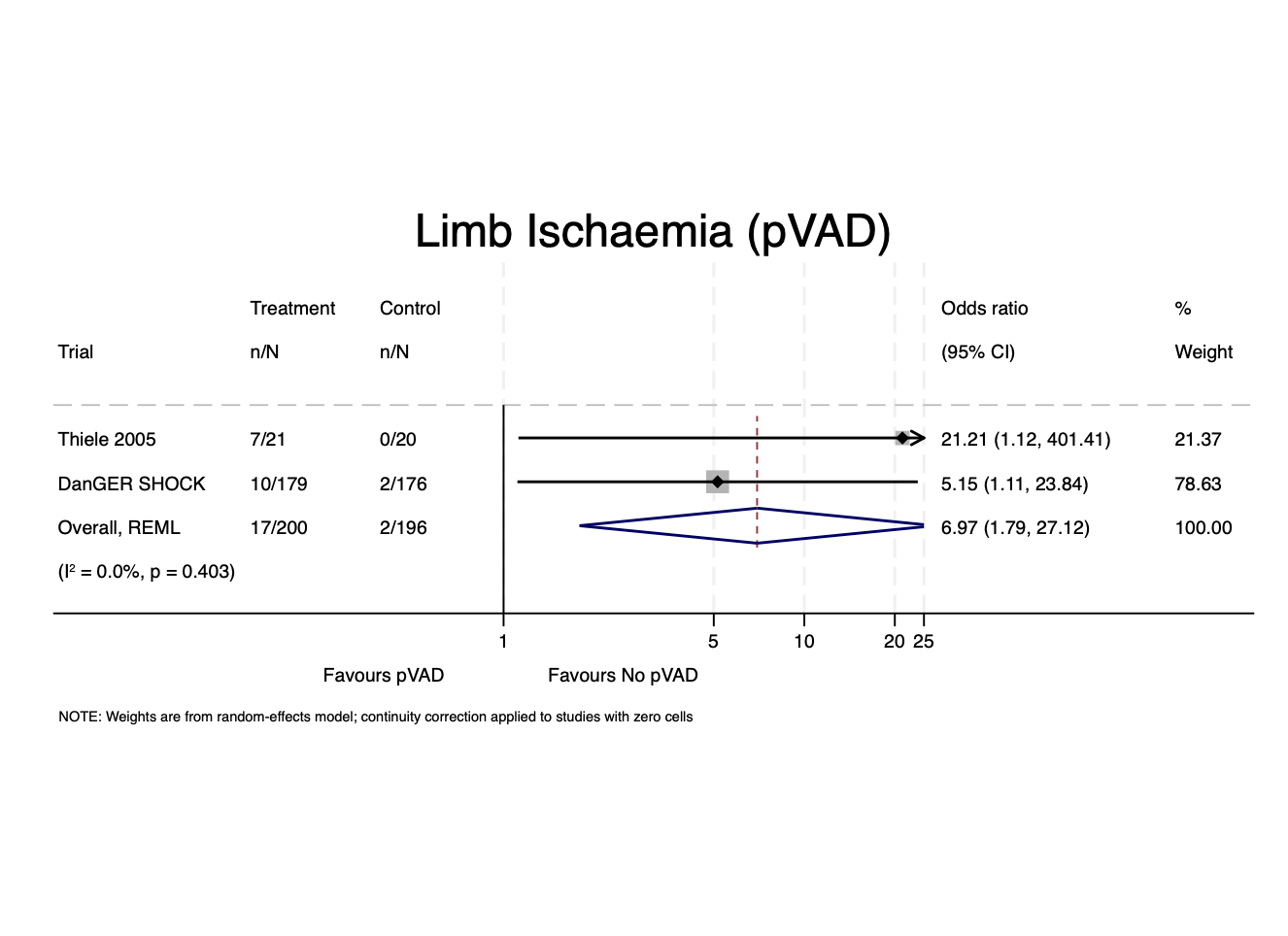


# Supplementary Figure 2. Limb Ischaemia (pVAD)

*pVAD* Percutaneous ventricular assist device, *CI* Confidence interval
